# Symmetry dictionary on charge and spin nonlinear responses for all magnetic point groups with nontrivial topological nature

**DOI:** 10.1093/nsr/nwad104

**Published:** 2023-04-27

**Authors:** Zhi-Fan Zhang, Zhen-Gang Zhu, Gang Su

**Affiliations:** School of Physical Sciences, University of Chinese Academy of Sciences, Beijing 100049, China; School of Physical Sciences, University of Chinese Academy of Sciences, Beijing 100049, China; School of Electronic, Electrical and Communication Engineering, University of Chinese Academy of Sciences, Beijing 100049, China; CAS Center for Excellence in Topological Quantum Computation, University of Chinese Academy of Sciences, Beijing 100190, China; School of Physical Sciences, University of Chinese Academy of Sciences, Beijing 100049, China; CAS Center for Excellence in Topological Quantum Computation, University of Chinese Academy of Sciences, Beijing 100190, China; Kavli Institute for Theoretical Sciences, University of Chinese Academy of Sciences, Beijing 100190, China

**Keywords:** nonlinear response, magnetic point group, matrix representation

## Abstract

Recently, charge or spin nonlinear transport with nontrivial topological properties in crystal materials has attracted much attention. In this paper, we perform a comprehensive symmetry analysis for all 122 magnetic point groups (MPGs) and provide a useful dictionary for charge and spin nonlinear transport from the Berry curvature dipole, Berry connection polarizability and Drude term with nontrivial topological nature. The results are obtained by conducting a full symmetry investigation of the matrix representations of six nonlinear response tensors. We further identify every MPG that can accommodate two or three of the nonlinear tensors. The present work gives a solid theoretical basis for an overall understanding of the second-order nonlinear responses in realistic materials.

## Teaser

A symmetry dictionary on charge and spin nonlinear responses from Berry curvature dipole, Berry connection polarizability and Drude term for all 122 magnetic point groups with nontrivial topological nature is elaborated.

## INTRODUCTION

The general matrix representations of a response tensor are very important to determine whether or not the corresponding physical phenomena exist in the materials with given point group symmetries, including, for instance, the linear Hall conductivity tensor in the anomalous Hall effect [1,2] and the magnetoelectric pseudo-tensor in the magnetoelectric effect (or Edelstein effect) [3]. In 2015, Sodemann and Fu [4] proposed a nonlinear Hall effect, being directly proportional to the dipole moment of the Berry curvature over occupied states, which is defined as a rank-two pseudo-tensor, the Berry curvature dipole (BCD), that is allowed in 18 classical point groups. This effect is in fact the second-order charge nonlinear Hall effect contributed by the BCD. Many materials such as bilayer or few-layer WTe_2_ [5–7], half-Heusler alloy CuMnSb [8], ferroelectric-like metal LiOsO_3_ [9] and so on [10–13] are found to accommodate this effect. Subsequently, Oiwa and Kusunose [14] proposed a systematic analysis for identifying essential parameters in various linear and nonlinear response tensors to decompose the response tensors into model-independent and model-dependent parts using the Keldysh formalism and the Chebyshev polynomial expansion method [15]. Another intrinsic contribution to the second-order charge nonlinear Hall effect has been proposed, which can be described by a rank-three tensor χ^int^ relating to the Berry connection polarizability (BCP) [16]. Recently, Liu *et al.* [17] showed the constraints of common point group operations on the in-plane tensor elements of the BCP, only including χ_*xyy*_ and χ_*yxx*_. The list of magnetic point groups (MPGs) classified by the existence or nonexistence of the second-order response tensor including the BCD, BCP and Drude effects is given in Table I of [18]. For second-order spin transport, there is the symmetric analysis under time-reversal symmetry (TRS) or space inversion [19,20], revealing that 16 magnetic point groups satisfying $\mathcal {PT}$ symmetry can make the second-order spin conductivity tensor nonzero [21] using the multipole classification [22].

All second-order charge and spin response tensors can be defined as rank-three tensors that are characterized by three 3 × 3 matrices. To the best of our knowledge, the theories mentioned above do not describe the second-order charge and spin effects in a compact form as a whole, nor matrix representations of the general tensors subjected to various MPGs. In this work, we present full matrix representations of the second-order charge and spin response tensors for all 122 MPGs. The powerful role of this symmetry analysis rests on the fact that the general second-order response effects, such as the BCD, BCP and Drude, can be determined directly from their MPGs whether they exist, coexist or do not exist at all. More importantly, we identify, for the first time, a contribution of BCP in addition to BCD and Drude for the second-order spin current. On all accounts the symmetry analyses are necessary for an overall understanding of topological nonlinear transport as well as searching candidate materials for hosting nonlinear responses of charge and spin.

## THE SECOND-ORDER CHARGE AND SPIN RESPONSE TENSORS

Under a driving electric field, we can obtain a second-order response tensor $\chi _{abc}^{(\sigma )}$ with the help of semi-classical Boltzmann transport theory under the relaxation-time approximation [23,24], which can be expressed as


**Figure 1.**
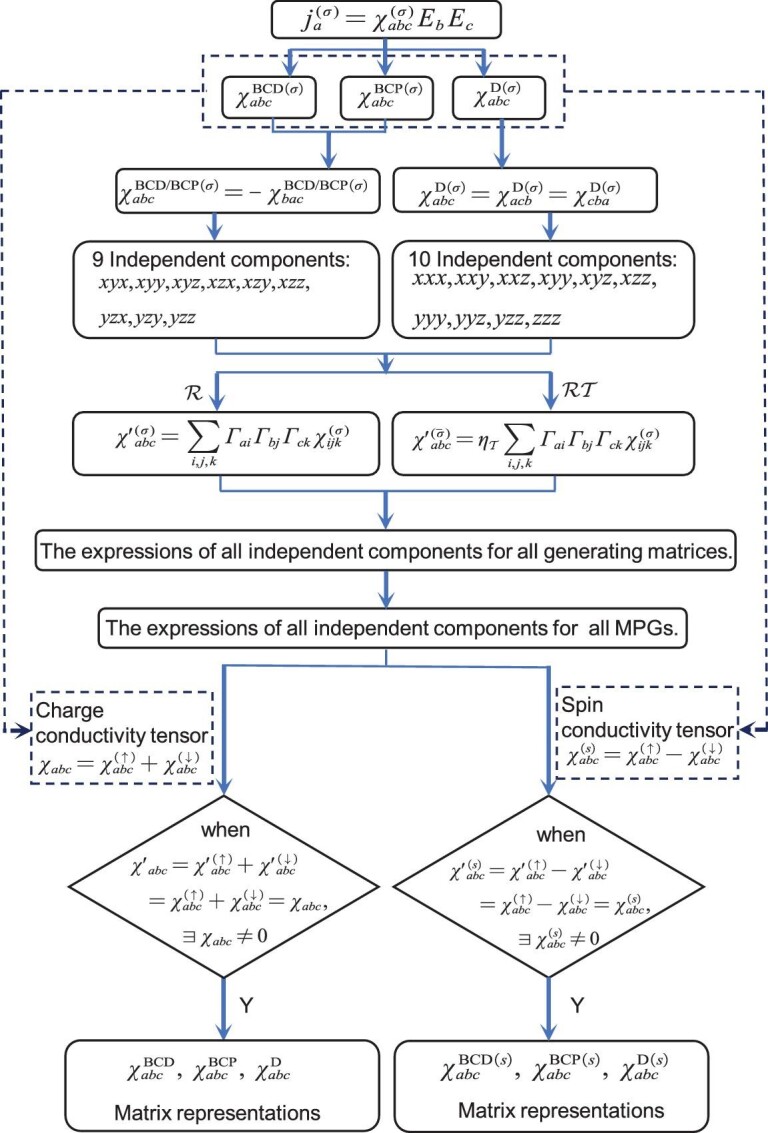
The calculation flow chart of matrix representations of second-order charge and spin response tensors.

(1) \begin{eqnarray*}
j_a^{(\sigma )}=\chi _{abc}^{(\sigma )}E_bE_c,
\end{eqnarray*}

(2) \begin{eqnarray*}
\chi _{abc}^{(\sigma )}=\chi _{abc}^{\text{BCD}(\sigma )}+\chi _{abc}^{\text{BCP}(\sigma )}+\chi _{abc}^{\text{D}(\sigma )},
\end{eqnarray*}

where *a, b, c* ∈ {*x, y*, ${z}$}, σ = ↑ or ↓ is the spin index of an electron and (1) implies the sum over the indices *b* and *c*. Equation (2) includes three different contributions, i.e. the BCD $\chi _{abc}^{\text{BCD}(\sigma )}$ [4], BCP $\chi _{abc}^{\text{BCP}(\sigma )}$ [17,18] and Drude $\chi _{abc}^{\text{D}(\sigma )}$ [25–28] terms for the second-order response. In the online supplementary data, we give detailed derivations and explicit expressions of these terms. Contributions $\chi _{abc}^{\text{BCP}(\sigma )}$ and $\chi _{abc}^{\text{BCD}(\sigma )}$ both have nine independent components because of the *a* ↔ *b* anti-symmetries [18,29], while for the Drude term $\chi _{abc}^{\text{D}(\sigma )}$, since any two indices are interchangeably symmetric, it has 10 independent components (shown in Fig. 1). Then the second-order charge current and the spin current can be expressed as $j_a =j_a^{(\uparrow )}+j_a^{(\downarrow )}$ and $j_a^{(s)}=(\hbar /2e)(j_a^{(\uparrow )}-j_a^{(\downarrow )})$, respectively [30]. Notably, the second-order charge response tensor can be written as $\chi _{abc}=\chi _{abc}^{\uparrow }+\chi _{abc}^{\downarrow }$, which has BCD, BCP and Drude terms, whereas the spin response tensor can be defined as $\chi _{abc}^{(s)}=\chi _{abc}^{\uparrow }-\chi _{abc}^{\downarrow }$. For convenience, we call these terms the spin-dependent BCD, BCP and Drude terms (χ^BCD(*s*)^, χ^BCP(*s*)^ and χ^D(*s*)^).

### CALCULATION PROCESS

In order to better describe magnetic materials, we introduce the magnetic point group (also called the Shubnikov group) $\mathcal {M}$, which can be categorized into three classes (see [31–34]).

Class I. MGP $\mathcal {M}$ does not contain the time-reversal operation $\mathcal {T}$.

Class II. MGP $\mathcal {M}$ contains $\mathcal {T}$ as an element.

Class III. MGP $\mathcal {M}$ contains $\mathcal {T}$ only in combination with another symmetry element $\mathcal {R}$, such as $\mathcal {C}_2^z\mathcal {T}$, where ${z}$ means that the axis of rotation is along the ${z}$ direction in Cartesian coordinates.

In three dimensions, there are 122 (32 + 32 + 58) MPGs, which are relisted in the online supplementary data. In order to facilitate sorting, all subsequent MPG symbols adopt international symbols [34]. The symbols of class-I MPGs do not contain the prime, all the symbols of class-II MPGs are suffixed by 1′ and class-III MPGs contain letters or numbers (except 1) plus the prime. It is then apparent that the class-I MPGs are equivalent to 32 classical point groups $\mathcal {G}$ ($\mathcal {R} \in \mathcal {G}$), which can be used to describe nonmagnetic crystals, or they correspond to the crystals in some magnetic states, where each atom has a certain magnetic moment, and neither $\mathcal {T}$ nor $\mathcal {RT}$ is a symmetric operation. Paramagnetic or diamagnetic materials with TRS can be described by class-II MPGs. Class III must correspond to magnetic crystals, such as ferromagnetic, antiferromagnetic or ferrimagnetic materials. In this work, we only focus on the case that can be covered by MPGs. For a general case accompanying spin-orbit coupling, the corresponding groups are beyond MPGs [35]. Since the case of MPGs is already complicated and should be the first step for a deeper understanding of the nonlinear topological transport from a symmetry aspect, it is better to make the study more specific to MPGs. For all symmetric operators in 122 MPGs, we can use 3 × 3 matrices to express them. Any set of symmetry matrices from which all symmetry matrices of a particular MPG may be obtained by multiplication are known as a set of generating matrices ${\Gamma}$ (see [33] and the online supplementary data), and, similarly, the corresponding symmetry operators are known as generators of the particular MPG. Then there are 18 generating matrices for all MPGs, which includes $\Gamma ^{n}$ and $\underline{\Gamma }^n = \Gamma ^n \mathcal {T},\, n \in \lbrace 1,2,\dots ,9\rbrace$ (see the online supplementary data).

For the second-order response tensors, the constraints from group symmetries on $\chi _{abc}^{(\sigma )}$ can be derived from


(3) \begin{eqnarray*}
\chi _{abc}^{(\sigma ^{\prime })} = \eta _{\mathcal {T}} \sum _{i,j,k} \Gamma _{ai}^n \Gamma _{bj}^n \Gamma _{ck}^n\chi _{ijk}^{(\sigma )},
\end{eqnarray*}

where $\Gamma _{pq}^n$ (*p* = *a, b, c*; *q* = *i, j, k*) represent the element in row *p* and column *q* of the generating matrix $\Gamma ^{n}$. The spin index σ′ = σ and $\eta _{\mathcal {T}} =1$ are only for class-I MPGs ($\mathcal {T}$ is not contained), while in class-II and class-III MPGs ($\mathcal {T}$ is contained), spin is reversed, i.e. $\sigma ^{\prime }=\bar{\sigma }$. Furthermore, the BCD term is even, $\chi _{abc}^{\text{BCD}(\sigma )}(\mathbf {k}) =\chi _{abc}^{\text{BCD}(\sigma )}(-\mathbf {k})$, with $\eta _{\mathcal {T}} = 1$, while the BCP and Drude terms are both odd with $\eta _{\mathcal {T}} = - 1$. Thus, we can derive the expressions with independent components for all MPGs in terms of the generating matrices (see the online supplementary data). As the components of charge and spin response tensors are unchanged before and after the symmetry operation, we can find every nonzero component of the matrices. To give a deeper understanding, we take the time-reversal operator as an example. Under $\mathcal {T}$, the transformation of charge conductivity terms can be expressed as


(4) \begin{eqnarray*}
\chi _{abc}^{\text{BCD}}({\bf k})\stackrel{\mathcal {T}}{\rightarrow} \chi _{abc}^{\text{BCD}}(-{\bf k})= \chi _{abc}^{\text{BCD}}({\bf k}),
\end{eqnarray*}

(5) \begin{eqnarray*}
\chi _{abc}^{\text{BCP/D}}({\bf k})\stackrel{\mathcal {T}}{\rightarrow} \chi _{abc}^{\text{BCP/D}}(-{\bf k})= -\chi _{abc}^{\text{BCP/D}}({\bf k}).
\end{eqnarray*}

According to (4), the BCD term is allowed, while the BCP and Drude terms must be zero in (5) for class-II MPGs. As shown in Fig. 1, we carry out similar calculations for other symmetric operations and we get all allowed MPGs and matrix representations of the corresponding tensors.

## SYMMETRY ANALYSIS

**Figure 2.**
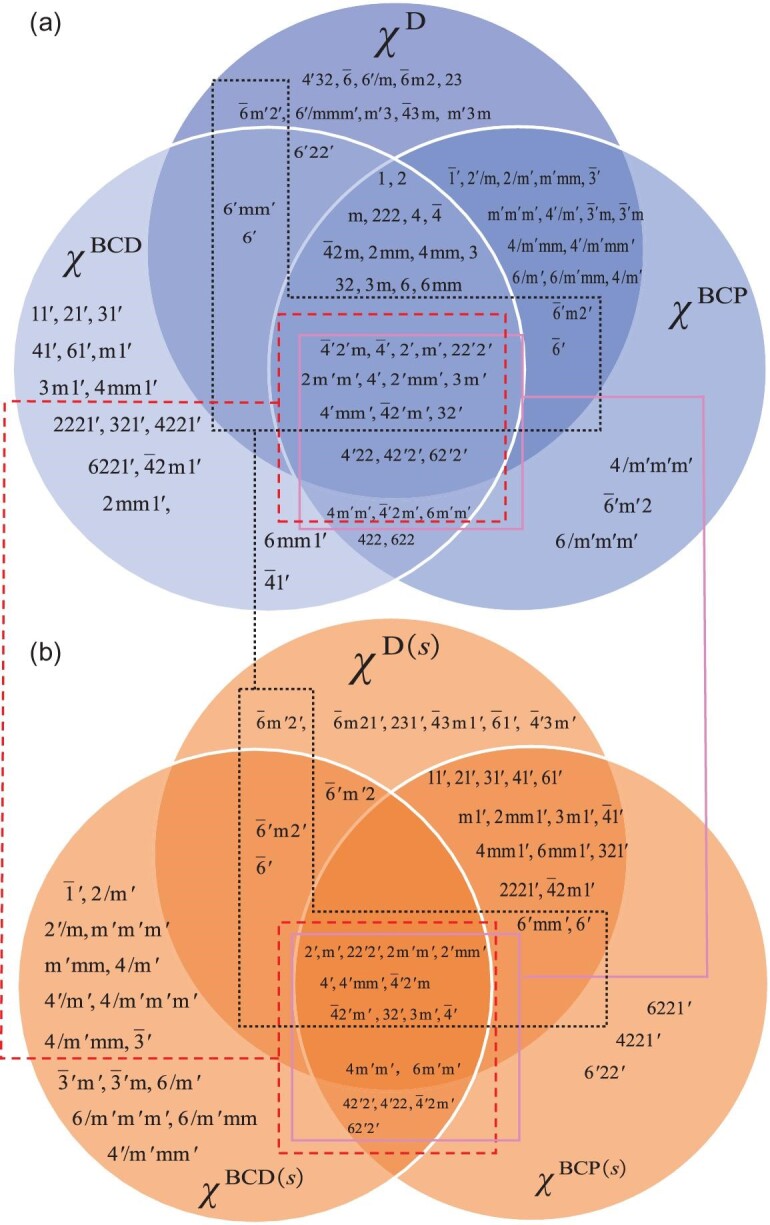
The classification and connection between MPGs allowing for (a) charge and (b) spin conductivity tensors. The dashed box connected by a dashed line represents MPGs where χ^BCD^ and χ^BCD(*s*)^ both exist, while the solid and dotted boxes represent MPGs where the BCP and Drude terms coexist.

Figure 2(a) shows all MPGs that allow for nonzero $\chi _{abc}^{\text{BCD}/\text{BCP}/\text{D}}$. There are 16 class-I MPGs that make $\chi _{abc}^{\text{BCD}}$ nonzero, which has been discussed in [4]. It should be noted that groups 23 (*T*) and 432 (*O*) restrict BCD to have only three equal diagonal components (similar to the magnetoelectric pseudo-tensor in [3]), whose corresponding second-order charge conductivity χ_*abc*_ has to be zero due to the *a* ↔ *b* anti-symmetry of the Levi-Civita symbol (see the online supplementary data).

Since the BCD term satisfies TRS, 16 class-II MPGs are found to accommodate χ^BCD^. In addition, there are another 21 class-III MPGs that encompass the nonzero BCD term. Because the BCP term is $\mathcal {T}$-odd, class-II MPGs cannot host the nonzero BCP term, whereas 16 class-I and 37 class-III MPGs can give nonzero BCP terms.

As the Drude term χ^D^ is symmetric when interchanging any two indices, the number of MPGs (allowing nonzero χ^D^) is a bit larger, namely, there are 18 class-I and 40 class-III MPGs. All these results are presented in Tables S3–S5 in the online supplementary data.

According to whether the three terms χ^BCD^, χ^BCP^ and χ^D^ (which are represented by three circles arranged counterclockwise) exist, coexist or not, we can divide the allowed MPGs into seven categories, as illustrated by different overlapping areas of three circles in Fig. 2(a). For instance, the lightest part of the lower left corner does not overlap the other two circles, implying that only χ^BCD^*is* nonzero in the 16 class-II MPGs of 11′, 21′, etc., because the BCD term is $\mathcal {T}$-even, but the BCP and Drude terms are $\mathcal {T}$-odd. By contrast, 4/m′m′m′, $\bar{6}^{\prime }\text{m}^{\prime }2$, 6/m′m′m′ make χ^BCP^ nonzero alone, and $\bar{6}$, 6′/m, etc. uniquely make χ^D^ nonzero. Furthermore, the overlapping region of any two circles represents that two of three terms can coexist but the remaining one is zero in some special MPGs. For example, some MPGs in classes I and III accommodate the coexistence of χ^BCD^ and χ^BCP^ (such as MPGs 422, 4m′m′), which is partly due to the fact that the two terms are nonzero in some combined operations (see [17]), e.g. $\mathcal {C}_2^x \mathcal {T}$ and $\mathcal {S}_4^x \mathcal {T}$. Fortunately, we can distinguish them by using different dependencies of the relaxation time in the low-frequency limit, i.e. χ^BCD^ ∝ τ [4], and χ^BCP^ ∝ τ^0^ [17,18]. In the experiments, the relaxation time τ depends on the electron concentration, which can be controlled by the disorder, doping or temperature.

The BCP and Drude terms are both nonzero only in 16 class-III MPGs, whereas the Drude term is proportional to τ^2^ in this situation. It is interesting to note that only three MPGs, 6′22′, 6′mm′, 6′, satisfy the condition that the BCD and Drude terms coexist. Of particular interest is the central part of Fig. 2(a) where there are 29 MPGs to host the coexistence of all three terms. Thus, we have an entire atlas of second-order charge nonlinear responses under different MPGs.

Since the spin degree of freedom plays an essential role in most materials, let us discuss the second-order spin response tensors for class-II and class-III MPGs; the results are given in Fig. 2(b). It turns out that all class-II MPGs forbid the spin-dependent BCD term because χ^BCD(↑/↓)^ becomes χ^BCD(↓/↑)^ in TRS, resulting in vanishing χ^BCD(*s*)^. Among 58 class-III MPGs, 37 of them accommodate the spin-dependent BCD contribution to the second-order spin current. There are 16 class-II and 21 class-III MPGs for χ^BCP(*s*)^ to be nonzero, while 18 class II and 21 class III for nonzero χ^D(*s*)^. What is so interesting is that the 37 allowed class-III MPGs for hosting χ^BCD(*s*)^ are identical to those for nonzero χ^BCP^ and the 21 allowed class-III MPGs for nonzero χ^BCP(*s*)^ are the same as those for hosting χ^BCD^. This important message is shown in Fig. 2 and we give a detailed description in the online supplementary data. In addition, we note here that the spin-dependent BCP term is proposed *for the first time*, which is also one of our central results.

We identify three categories in which the spin-dependent BCD, BCP and Drude terms exist uniquely (no coexistence), and there are 16, 3 and 6 MPGs, respectively. Moreover, there are four MPGs ($42^{\prime }2^{\prime }, 4^{\prime }22, \bar{4}^{\prime }2\text{m}^{\prime }$ and 62′2′) allowing coexistence of the spin-dependent BCD and BCP. In 11′, 21′, etc., the second-order spin currents are contributed by both the spin-dependent BCP and Drude contributions; however, only three MPGs, $\bar{6}^{\prime }\text{m}^{\prime }2$, $\bar{6}^{\prime }\text{m}2^{\prime }$ and $\bar{6}^{\prime }$, allow for the coexistence of the spin-dependent BCD and Drude terms when BCP has no contribution. Similarly, the central part of Fig. 2(b) shows that the three terms are all nonzero in 14 class-III MPGs. All these allowed class-II and class-III MPGs are shown in Tables S6–S8 in the online supplementary data.

Importantly, we show in Fig. 2 whether the second-order charge and spin currents can appear simultaneously or not. The dashed boxes indicate that, for the MPGs that belong to class III, χ^BCD^ and χ^BCD(*s*)^ both exist. For example, for MPG m′, the generating matrices are $\Gamma ^0$ and $\underline{\Gamma }^5$ ($=\Gamma ^5\mathcal {T}$). The former is a unitary matrix and the latter represents the operator $\mathcal {P}\mathcal {C}_2^z \mathcal {T}$, which makes the charge and spin response tensors all nonzero. In addition, MPGs outside the boxes do not allow coexistence of BCD and spin-dependent BCD; only one of them is allowed, such as MPG 6′ that allows χ^BCD^ but forbids χ^BCD(*s*)^ owing to the combination of generating matrices $\Gamma ^6$ ($=\mathcal {C}_3^{1z}$) and $\underline{\Gamma }^3$ ($=\Gamma ^3 \mathcal {T} = \mathcal {C}_2^z \mathcal {T}$). MPGs containing $\mathcal {PT}$ (for example, $\bar{1}^{\prime }, 2/\text{m}^{\prime }$) only have nonzero χ^BCD(*s*)^ (but zero χ^BCP(*s*)^ and χ^D(*s*)^), which reproduces the results in [21]. Furthermore, the nonzero χ^BCD(*s*)^ may be tested in many collinear magnets, such as LiFePO_4_ [36] and Cr_2_O_3_ [37]. The solid boxes encircle 18 MPGs that allow for the coexistence of second-order charge and spin currents from the BCP term. Outside the solid boxes are incompatible MPGs. The spin-dependent BCP term (zero charge BCP) can exist in 19 MPGs including 16 class-II and three class-III MPGs. In particular, three class-III MPGs (6′22′, 6′mm′ and 6′) may be interesting since they host magnetic materials that may have wide spin applications. The dotted boxes show the nonzero Drude term in second-order charge and spin responses. Outside the dotted boxes we find four class-II MPGs ($\bar{6}\text{m}21^{\prime }, 231^{\prime }, \bar{4}3\text{m}1^{\prime }$ and $\bar{6}1^{\prime }$) and one class-III MPG ($\bar{4}^{\prime }3\text{m}^{\prime }$) in which only χ^D(*s*)^ is nonzero but the others (χ^BCD/BCP/D^ and χ^BCD(s)/BCP(s)^) are all zero. We note that these five MPGs are of particular interest as materials with these MPGs can be ideal platforms to study spin transport without involving charge transport.

## MATRIX REPRESENTATION

Besides the symmetry analysis, it is still necessary to obtain the specific matrix representations of the second-order charge and spin response tensors, χ_*abc*_ and $\chi _{abc}^{(s)}$, for all allowed MPGs (see the online supplementary data). Here we give two examples in Table 1. Let us first study MPG m1′ (1′ indicates that the system satisfies TRS) in bilayer or few-layer WTe_2_ [40]. For the BCD term in the second-order charge response tensor, the nonzero independent components are *xyx, xyy, xzz, yzz*. In two or quasi-two dimensions, the driving electric field and the response current are both in the *x*-*y* plane, m (i.e. the C_1${v}$_ group) is the only MPG that has nonzero components (see the online supplementary data) and the maximal symmetry in which a second-order current exists is the single mirror line under TRS [4]. (In two dimensions, the components of the charge response tensor are not zero for MPG m, which only has a mirror symmetric operator; hence, the maximum symmetry that exists for a second-order charge current is a single mirror line.) In this case the second-order response charge current contributed by BCD is a transverse effect, being perpendicular to the direction of the external electric field. In addition, many MPGs contain the combination of spatial and time-reversal symmetry ($\mathcal {PT}$), which forbids the BCD contribution but allows for the BCP and Drude terms, such as MPG $2'/\text{m}$ in antiferromagnetic tetragonal CuMnAs [18] as another example. According to Table 1, the BCP term only has nonzero independent elements *xyx, xyy, xzz, yzz*, while the Drude term has *xxx, xxy, xyy, xzz, yyy, yzz*. Intriguingly, this suggests that the second-order charge current contributed by BCP is a pure transverse effect, but the Drude term can manifest itself as transverse and longitudinal components. The matrix representations for the second-order charge (Tables S9–S14) and spin (Tables S15–S19) response tensors of all MPGs can be found in the online supplementary data.

**Table 1.** The nonzero independent components and candidate materials of the charge response tensor for MPGs m1′ and 2′/m. The asterisk indicates that the nonlinear current has been reported.

MPG	$\chi _{abc}^{\text{BCD}}$	$\chi _{abc}^{\text{BCP}}$	$\chi _{abc}^{\text{D}}$	Candidate materials
m1′	*xyx, xyy, xzz*	Zero	Zero	WTe$_2 ^{*}$, Na_2_MnF_5_ [38]
	*yzz*			
2′/m	Zero	*xyx, xyy, xzz*,	*xxx, xxy, xyy*	CuMnAs*, ErGe_3_ [39]
		*yzz*	*xzz, yyy, yzz*	

## MORE MATERIALS

In addition to the materials mentioned above, we can inspect the databases [41–47] or existing predicted structures [48–50] to find corresponding materials for every MPG hosting second-order nonlinear charge and spin responses. On the other hand, we can also perform numerical calculations through known *k* · *p* models [51,52] or first-principles calculations to find more materials with giant second-order effects. In the online supplementary data, we present corresponding materials (such as the last column in Table 1) for each class of MPGs, where the asterisk in the upper right corner indicates that relevant theoretical or experimental results have been reported.

## SYMMETRY

In this work, we show that both the second-order charge and spin response currents have three contributions, i.e. the BCD, BCP and Drude terms, all of which can be written as rank-three tensors. Besides, a new term, i.e. the spin-dependent BCP tensor in the second-order spin response, is proposed in this work. To discuss the second-order nonlinear charge and spin responses, a detailed symmetry analysis and matrix representations of six nonlinear response tensors are given for 122 MPGs. We present a symmetry dictionary to determine whether the second-order charge and spin response tensors can exist, coexist or not for a given MPG, and a few candidate magnetic materials are also suggested to detect the specific second-order nonlinear responses. Here we should point out that, although this work does not include the scattering or electron-phonon interaction effects, some recent developments [53–57] make good complementary contributions to this intriguing topic, which, together with this present work, provide a comprehensive understanding of the second-order nonlinear charge and spin responses in realistic materials. Our symmetry analysis is also applicable to AC measurements, which can determine the response tensors by a lock-in amplifier. Although the present work only considers intra-band transitions, it may be a useful reference for further explorations, comparisons and inspirations, for example nonlinear optical measurements (such as the second harmonic generation).

## Supplementary Material

nwad104_Supplemental_File

## References

[1] Grimmer H . General relations for transport properties in magnetically ordered crystals. Acta Crystallogr A 1993; 49: 763. 10.1107/S0108767393003770

[2] Seemann M , Ködderitzsch D, Wimmer S et al. Symmetry-imposed shape of linear response tensors. Phys Rev B 2015; 92: 155138. 10.1103/PhysRevB.92.155138

[3] He WY , Law KT. Magnetoelectric effects in gyrotropic superconductors. Phys Rev Res 2020; 2: 012073. 10.1103/PhysRevResearch.2.012073

[4] Sodemann I , Fu L. Quantum nonlinear Hall effect induced by Berry curvature dipole in time-reversal invariant materials. Phys Rev Lett 2015; 115: 216806. 10.1103/PhysRevLett.115.216806 26636867

[5] Du ZZ , Wang CM, Lu HZ et al. Band signatures for strong nonlinear Hall effect in bilayer WTe_2_. Phys Rev Lett 2018; 121: 266601. 10.1103/PhysRevLett.121.266601 30636120

[6] Ma Q , Xu SY, Shen H et al. Observation of the nonlinear Hall effect under time-reversal-symmetric conditions. Nature 2019; 565: 337–42. 10.1038/s41586-018-0807-6 30559379

[7] Kang K , Li T, Sohn E et al. Nonlinear anomalous Hall effect in few-layer WTe_2_. Nat Mater 2019; 18: 324–8. 10.1038/s41563-019-0294-7 30804510

[8] Shao DF , Zhang SH, Gurung G et al. Nonlinear anomalous Hall effect for Néel vector detection. Phys Rev Lett 2020; 124: 067203. 10.1103/PhysRevLett.124.067203 32109084

[9] Xiao RC , Shao DF, Huang W et al. Electrical detection of ferroelectriclike metals through the nonlinear Hall effect. Phys Rev B 2020; 102: 024109. 10.1103/PhysRevB.102.024109

[10] Zhang CP , Xiao J, Zhou BT et al. Giant nonlinear Hall effect in strained twisted bilayer graphene. Phys Rev B 2022; 106: L41111. 10.1103/PhysRevB.106.L041111

[11] Battilomo R , Scopigno N, Ortix C. Berry curvature dipole in strained graphene: a Fermi surface warping effect. Phys Rev Lett 2019; 123: 196403. 10.1103/PhysRevLett.123.196403 31765194

[12] He Z , Weng H. Giant nonlinear Hall effect in twisted bilayer WTe_2_. npj Quantum Mater 2021; 6: 101. 10.1038/s41535-021-00403-9

[13] Chen C , Wang H, Wang D et al. Strain-engineered nonlinear Hall effect in HgTe. SPIN 2019; 09: 1940017. 10.1142/S2010324719400174

[14] Oiwa R , Kusunose H. Systematic analysis method for nonlinear response tensors. J Phys Soc Jpn 2022; 91: 014701. 10.7566/JPSJ.91.014701

[15] João SM , Lopes JMVP. Basis-independent spectral methods for non-linear optical response in arbitrary tight-binding models. J Phys Condens Matter 2019; 32: 125901. 10.1088/1361-648X/ab59ec 31751952

[16] Gao Y , Yang SA, Niu Q. Field induced positional shift of Bloch electrons and its dynamical implications. Phys Rev Lett 2014; 112: 166601. 10.1103/PhysRevLett.112.166601 24815661

[17] Liu H , Zhao J, Huang YX et al. Intrinsic second-order anomalous Hall effect and its application in compensated antiferromagnets. Phys Rev Lett 2021; 127: 277202. 10.1103/PhysRevLett.127.277202 35061417

[18] Wang C , Gao Y, Xiao D. Intrinsic nonlinear Hall effect in antiferromagnetic tetragonal CuMnAs. Phys Rev Lett 2021; 127: 277201. 10.1103/PhysRevLett.127.277201 35061403

[19] Hamamoto K , Ezawa M, Kim KW et al. Nonlinear spin current generation in noncentrosymmetric spin-orbit coupled systems. Phys Rev B 2017; 95: 224430. 10.1103/PhysRevB.95.224430

[20] Zhang ZF , Zhu ZG, Su G. Theory of nonlinear response for charge and spin currents. Phys Rev B 2021; 104: 115140. 10.1103/PhysRevB.104.115140

[21] Hayami S , Yatsushiro M, Kusunose H. Nonlinear spin Hall effect in *PT*-symmetric collinear magnets. Phys Rev B 2022; 106: 024405. 10.1103/PhysRevB.106.024405

[22] Yatsushiro M , Kusunose H, Hayami S. Multipole classification in 122 magnetic point groups for unified understanding of multiferroic responses and transport phenomena. Phys Rev B 2021; 104: 054412. 10.1103/PhysRevB.104.054412

[23] Mahan GD . Many-Particle Physics, 3rd edn. New York: Kluwer Acdemic, 2000.

[24] Abrikosov AA . Fundamentals of the Theory of Metals. Amsterdam: North-Holland, 1988.

[25] Gao Y . Semiclassical dynamics and nonlinear charge current. Front Phys 2019; 14: 33404. 10.1007/s11467-019-0887-2

[26] Železný J , Fang Z, Olejník K et al. Unidirectional magnetoresistance and spin-orbit torque in NiMnSb. Phys Rev B 2021; 104: 054429. 10.1103/PhysRevB.104.054429

[27] Itahashi YM , Ideue T, Hoshino S et al. Giant intrinsic rectification and nonlinear Hall effect under time-reversal symmetry in a trigonal superconductor, arXiv, 2022, preprint: not peer reviewed. https://arxiv.org/abs/2202.11876 10.1038/s41467-022-29314-4 PMC8964720 35351870

[28] Lesne E , Sağlam YG, Battilomo R et al. Designing spin and orbital sources of Berry curvature at oxide interfaces. Nat Mater 2023; 22: 576–82. 10.1038/s41563-023-01498-0 36928382 PMC10156604

[29] Du ZZ , Wang CM, Sun HP et al. Quantum theory of the nonlinear Hall effect. Nat Commun 2021; 12: 5038. 10.1038/s41467-021-25273-4 34413295 PMC8377135

[30] Kane CL , Mele EJ. Quantum spin Hall effect in graphene. Phys Rev Lett 2005; 95: 226801. 10.1103/PhysRevLett.95.226801 16384250

[31] Shubnikov A , Belov N. Colored Symmetry. Oxford: Pergamon, 1964.

[32] Newnham RE . Properties of Materials: Anisotropy, Symmetry, Structure. New York: Oxford University Press, 2005.

[33] Birss RR . Macroscopic symmetry in space-time. Rep Prog Phys 1963; 26: 307. 10.1088/0034-4885/26/1/309

[34] El-Batanouny M , Wooten F. Symmetry and Condensed Matter Physics: A Computational Approach. Cambridge: Cambridge University Press, 2008.

[35] Liu P , Li J, Han J et al. Spin-group symmetry in magnetic materials with negligible spin-orbit coupling. Phys Rev X 2022; 12: 021016. 10.1103/PhysRevX.12.021016

[36] Toft-Petersen R , Reehuis M, Jensen TBS et al. Anomalous magnetic structure and spin dynamics in magnetoelectric LeFePO_4_. Phys Rev B 2015; 92: 024404. 10.1103/PhysRevB.92.024404

[37] Brown PJ , Forsyth JB, Lelièvre-Berna E et al. Determination of the magnetization distribution in Cr_2_O_3_ using spherical neutron polarimetry. J Phys Condens Matter 2002; 14: 1957–66. 10.1088/0953-8984/14/8/323

[38] Núñez P , Roisnel T, Tressaud A. Magnetic structure of the chain mn(III) fluoride Na_2_MnF_5_. Solid State Commun 1994; 92: 601–5. 10.1016/0038-1098(94)00536-2

[39] Schobinger-Papamantellos P , André G, Rodríguez-Carvajal J et al. The magnetic ordering of the novel compound ErGe_3_. J Alloys Compd 1996; 232: 165–8. 10.1016/0925-8388(95)01949-9

[40] Brown BE . The crystal structures of WTe_2_ and high-temperature MoTe_2_. Acta Crystallogr 1966; 20: 268–74. 10.1107/S0365110X66000513

[41] Bilbao Crystallographic Server . https://www.cryst.ehu.es/.

[42] Gallego SV , Perez-Mato JM, Elcoro L et al. MAGNDATA: towards a database of magnetic structures. I. The commensurate case. J Appl Cryst 2016; 49: 1750–76. 10.1107/S1600576716012863

[43] SV Gallego , Perez-Mato JM, Elcoro L et al. MAGNDATA: towards a database of magnetic structures. II. The incommensurate case. J Appl Cryst. 2016; 49: 1941–56. 10.1107/S1600576716015491

[44] Topological Magnetic Materials Database . https://www.topologicalquantumchemistry.fr/magnetic/.

[45] Bradlyn B , Elcoro L, Cano J et al. Topological quantum chemistry. Nature 2017;547:298–305. 10.1038/nature23268 28726818

[46] Vergniory MG , Elcoro L, Felser C et al. A complete catalogue of high-quality topological materials. Nature 2019; 566: 480–5. 10.1038/s41586-019-0954-4 30814710

[47] Elcoro L , Wieder BJ, Song Z et al. Magnetic topological quantum chemistry. Nat Commun 2021; 12: 5965. 10.1038/s41467-021-26241-8 PMC8514474 34645841

[48] Watanabe H , Yanase Y. Group-theoretical classification of multipole order: emergent responses and candidate materials. Phys Rev B 2018; 98: 245129. 10.1103/PhysRevB.98.245129

[49] Xu Y , Elcoro L, Song ZD et al. High-throughput calculations of magnetic topological materials. Nature 2020; 586: 702–7. 10.1038/s41586-020-2837-0 33116291

[50] Călugăru D , Chew A, Elcoro L et al. General construction and topological classification of crystalline flat bands. Nat Phys 2021; 18: 185–9. 10.1038/s41567-021-01445-3

[51] Jiang Y , Fang Z, Fang C. A k · p effective Hamiltonian generator. Chin Phys Lett 2021; 38: 077104. 10.1088/0256-307X/38/7/077104

[52] Tang F , Wan X. Exhaustive construction of effective models in 1651 magnetic space groups. Phys Rev B 2021; 104: 085137. 10.1103/PhysRevB.104.085137

[53] Watanabe H , Yanase Y. Nonlinear electric transport in odd-parity magnetic multipole systems: application to mn-based compounds. Phys Rev Res 2020; 2: 043081. 10.1103/PhysRevResearch.2.043081

[54] Michishita Y , Nagaosa N. Dissipation and geometry in nonlinear quantum transports of multiband electronic systems, Phys Rev B 2022; 106: 125114. 10.1103/PhysRevB.106.125114

[55] Watanabe H , Yanase Y. Chiral photocurrent in parity-violating magnet and enhanced response in topological antiferromagnet. Phys Rev X 2021; 11: 011001. 10.1103/PhysRevX.11.011001

[56] Wang YD , Zhang ZF, Zhu ZG et al. An intrinsic non-Hall-type nonlinear current, arXiv, 2023, preprint: not peer reviewed. https://arxiv.org/abs/2207.01182

[57] Lahiri S , Das K, Culcer D et al., Intrinsic nonlinear conductivity induced by the quantum metric, arXiv, 2023, preprint: not peer reviewed. https://arxiv.org/abs/2207.02178

