# Supplementary material for: Symmetry dictionary on charge and spin nonlinear responses for all magnetic point groups with nontrivial topological nature
Source: Natl Sci Rev. 2023 Apr 27;10(11):nwad104. doi: 10.1093/nsr/nwad104 (PMC10561712; doi:10.1093/nsr/nwad104)
Supplement: nwad104_Supplemental_File [file nwad104_supplemental_file.pdf]

Supplementary Data for
"Symmetry Dictionary on Charge and Spin Nonlinear Responses for All Magnetic Point Groups with Nontrivial Topological Nature"

Zhi-Fan Zhang[1], Zhen-Gang Zhu[1,2,3,*] and Gang Su[4,1,3,†]

[1] *School of Physical Sciences, University of Chinese Academy of Sciences, Beijing 100049, China.*

[2] *School of Electronic, Electrical and Communication Engineering, University of Chinese Academy of Sciences, Beijing 100049, China.*

[3] *CAS Center for Excellence in Topological Quantum Computation, University of Chinese Academy of Sciences, Beijing 100049, China.*

[4] *Kavli Institute for Theoretical Sciences, University of Chinese Academy of Sciences, Beijing 100190, China.*

CONTENTS

SI. The Second Charge and Spin Response Tensors 2

SII. The Introduction of 122 magnetic point groups 4

SIII. Symmetry analysis 7
A. The second-order charge response tensor 7
B. The second-order spin response tensor 9
C. Two-dimensional matrix representations for all response tensors 22

References 26

SI. THE SECOND CHARGE AND SPIN RESPONSE TENSORS

We suppose that there is an ac driving electric field $\mathscr{E}_c(t) = \mathrm{Re}\{E_c e^{i\omega t}\}(c = x, y, z)$ and in the relaxation-time approximation, the Boltzmann equation reads

$$-\frac{e\tau}{\hbar}\mathscr{E}_c\partial_c f + \tau\partial_t f = f_0 - f, \tag{S1}$$

where f_0 is the equilibrium distribution in the absence of $\mathscr{E}_c(t)$, τ is the relaxation time of electrons, $\partial_c = \frac{\partial}{\partial k_c}$, and $\partial_t = \frac{\partial}{\partial t}$. Here we write the infinite-order distribution function like this: $f = \mathrm{Re}\{f_0 + f_1 + f_2\}$, we have [1]

$$\begin{aligned} f_1 &= \frac{e\tau E_c \partial_c f_0}{\hbar(1+i\omega\tau)} e^{i\omega t}, \\ f_2 &= \frac{e^2\tau^2 E_c E_d \partial_{cd} f_0}{2\hbar(1+i\omega\tau)(1+2i\omega\tau)} + \frac{e^2\tau^2 E_c^* E_d \partial_{cd} f_0}{2\hbar(1+i\omega\tau)(1+2i\omega\tau)} e^{2i\omega t}. \end{aligned} \tag{S2}$$

The response charge current density is given by

$$j_a = -e\int_k v_a f, \tag{S3}$$

where $\int_k = \int \frac{d^3k}{(2\pi)^3}$, v_a is the electric velocity in the a direction, and we omit the band index. Gao *et al.* [2] proposed a modification of Berry curvature under driving electric field,

$$\dot{\boldsymbol{r}} = \frac{1}{\hbar}\frac{\partial \varepsilon}{\partial \boldsymbol{k}} - \dot{\boldsymbol{k}} \times [\nabla \times (\mathbf{A} + \mathbf{A}')], \tag{S4}$$

$$\dot{k} = -\frac{e}{\hbar}\mathbf{E}, \tag{S5}$$

where $\mathbf{A}$ is the Berry curvature connection, the modification is $A'_a = eG_{ac}\mathcal{E}_c$. $G^n_{ac}(\boldsymbol{k}) = 2\mathrm{Re}\sum_{m\neq n} \frac{\mathcal{A}^{nm}_a(\boldsymbol{k})\mathcal{A}^{mn}_c(\boldsymbol{k})}{\varepsilon_n(\boldsymbol{k}) - \varepsilon_m(\boldsymbol{k})}$ is the Berry connection poloarizability (BCP). Here we can directly get the electric velocity in the a direction,

$$v_a = \frac{1}{\hbar}\frac{\partial \varepsilon}{\partial k_a} + \frac{e}{\hbar}\epsilon_{abd}\mathcal{E}_b\Omega_d + \frac{e^2}{\hbar}(\partial_a G_{bc} - \partial_b G_{ac})\mathcal{E}_b\mathcal{E}_c, \tag{S6}$$

where ϵ_{abc} is the Levi-Civita symbol and the last term is the modification. Thus, we can get the response charge current $j_a = \mathrm{Re}\{j_a^0 + j_a^1 + j_a^2\}$,

$$\begin{aligned}
j_a^0 =& -\frac{e}{\hbar}\int_k \frac{\partial \varepsilon}{\partial k_a} f_0,\\
j_a^1 =& -\left[\frac{e^2\tau}{2\hbar^2(1+i\omega\tau)}\int_k \frac{\partial \varepsilon}{\partial k_a}\frac{\partial f_0}{\partial k_b} + \frac{e^2}{\hbar}\epsilon_{abd}\int_k \Omega_d f_0\right] E_b e^{i\omega t},\\
j_a^2 =& -\frac{e^3\tau^2}{2\hbar^3}\left[\frac{E_b E_c}{(1+i\omega t)(1+2i\omega t)}e^{2i\omega t} + \frac{E_b^* E_c}{(1+i\omega t)}\right]\int_k \frac{\partial \varepsilon}{\partial^2 k_a}\frac{\partial f_0}{\partial k_b \partial k_c}\\
& -\frac{e^3\tau}{2\hbar^2}\epsilon_{abd}\left[\frac{E_b E_c e^{2i\omega t} + E_b^* E_c}{1+i\omega t}\right]\int_k \Omega_d \frac{\partial f_0}{\partial k_c}\\
& -\frac{e^3}{2\hbar}\left(E_b E_c e^{i\omega t} + E_b^* E_c\right)\int_k \left(\partial_a G_{bc} - \partial_b G_{ac}\right) f_0,
\end{aligned} \tag{S7}$$

where j_a^2 refers to the second-order current in the direction of a in response to the driving electric field, which is the part of our work. In the low frequency limit,

$$\begin{aligned}
j_a^2\,(\omega \to 0) &= \chi_{abc} E_b E_c,\\
\chi_{abc}^{\mathrm{BCD}} &= -\frac{e^3\tau}{\hbar^2}\int_k \epsilon_{abd}\Omega_d \frac{\partial f_0}{\partial k_c},\\
\chi_{abc}^{\mathrm{BCP}} &= -\frac{e^3}{\hbar}\int_k \left(\partial_a G_{bc} - \partial_b G_{ac}\right) f_0,\\
\chi_{abc}^{\mathrm{D}} &= -\frac{e^3\tau^2}{\hbar^3}\int_k \frac{\partial \varepsilon}{\partial k_a}\frac{\partial^2 f_0}{\partial k_b \partial k_c}.
\end{aligned} \tag{S8}$$

Therefore, the dependence of the coeffficient on τ is

$$\chi_{abc}^{\mathrm{BCD}} \propto \tau, \qquad \chi_{abc}^{\mathrm{BCP}} \propto \tau^0, \qquad \chi_{abc}^{\mathrm{D}} \propto \tau^2. \tag{S9}$$

Here we figure out the second-order charge response coefficient is a rank-three tensor, contributing by the three parts, BCD [1] , BCP [3, 4] and the Drude [5, 6]. According to the Ref. [6], we can do some further calculations, and we have

$$\chi_{abc}^{\mathrm{D}} = -\frac{e^3\tau^2}{2\hbar^3}\int_k v_a v_b v_c \frac{\partial^2 f_0}{\partial \varepsilon^2}. \tag{S10}$$

Further, if we take the electric spin into account, the second-order conductivity can be expressed as

$$j_a^{2(\sigma)} = \chi_{abc}^{(\sigma)} E_b E_c, \tag{S11}$$

$$\chi_{abc}^{(\sigma)} = \chi_{abc}^{\mathrm{BCD}(\sigma)} + \chi_{abc}^{\mathrm{BCP}(\sigma)} + \chi_{abc}^{\mathrm{D}(\sigma)}, \tag{S12}$$

where the $\sigma = \uparrow$ or $\downarrow$ is spin index of electrons. Then the second-order charge current and the spin current can be expressed separately as $j_a = j_a^{(\uparrow)} + j_a^{(\downarrow)}$ and $j_a^{(s)} = (\hbar/2e)(j_a^{(\uparrow)} - j_a^{(\downarrow)})$ [7]. Hence, we can write the second-order charge and spin response tensors,

$$\chi_{abc} = \chi_{abc}^{\mathrm{BCD}} + \chi_{abc}^{\mathrm{BCP}} + \chi_{abc}^{\mathrm{D}}, \tag{S13}$$

$$\chi_{abc}^{(s)} = \chi_{abc}^{\mathrm{BCD}(s)} + \chi_{abc}^{\mathrm{BCP}(s)} + \chi_{abc}^{\mathrm{D}(s)}, \tag{S14}$$

where $\chi_{abc} = \chi_{abc}^{\uparrow} + \chi_{abc}^{\downarrow}$, $\chi_{abc}^{(s)} = \chi_{abc}^{\uparrow} - \chi_{abc}^{\downarrow}$, and we use the upper index s to represent the spin response.

These three terms are the major contributions of the second-order response. Take the charge response as example, for the BCD and BCP terms they all have $a \leftrightarrow b$ anti-symmetries, which have the same 9 independent components,

$$\chi_x^{\mathrm{BCD/BCP}} = \begin{pmatrix} 0 & 0 & 0\\ xyx & xyy & xyz\\ xzx & xzy & xzz \end{pmatrix}, \chi_y^{\mathrm{BCD/BCP}} = \begin{pmatrix} -xyx & -xyy & -xyz\\ 0 & 0 & 0\\ yzx & yzy & yzz \end{pmatrix}, \chi_z^{\mathrm{BCD/BCP}} = \begin{pmatrix} -xzx & -xzy & -xzz\\ -yzx & -yzy & -yzz\\ 0 & 0 & 0 \end{pmatrix}. \tag{S15}$$

However the Drude term have the interchangeably symmetric for any two indices, and have 10 independent components,

$$\chi_x^{\mathrm{D}} = \begin{pmatrix} xxx & xxy & xxz \\ xxy & xyy & xyz \\ xxz & xyz & xzz \end{pmatrix}, \chi_y^{\mathrm{D}} = \begin{pmatrix} xxy & xyy & xyz \\ xyy & yyy & yyz \\ xyz & yyz & yzz \end{pmatrix}, \chi_z^{\mathrm{D}} = \begin{pmatrix} xxz & xyz & xzz \\ xyz & yyz & yzz \\ xzz & yzz & zzz \end{pmatrix}. \tag{S16}$$

We can get similar results for the second-order spin response tensors.

SII. THE INTRODUCTION OF 122 MAGNETIC POINT GROUPS

The magnetic point groups(MPGs) $\mathcal{M}$, or we can call the Shubnikov groups can be divided into three classes, the number of which are 32, 32 and 58. In Table S1, we give the international symbols and Schoenfies symbols for the Class I, as well as the international symbols of the Class II and III. Next we figure out all the production matrices (generators) of the three classes of magnetic point groups, which are represented by the symbol $\mathit{\Gamma}^i, i = 0, 1, 2, 3, ..., 9$ and $\underline{\mathit{\Gamma}^k} = \mathit{\Gamma}^k \mathcal{T}, k = 0, 1, 2, 3, ..., 9,$

$$\mathit{\Gamma}^1 = \mathcal{P} = \begin{pmatrix} -1 & 0 & 0 \\ 0 & -1 & 0 \\ 0 & 0 & -1 \end{pmatrix}, \qquad \mathit{\Gamma}^2 = \mathcal{C}_2^y = \begin{pmatrix} -1 & 0 & 0 \\ 0 & 1 & 0 \\ 0 & 0 & -1 \end{pmatrix}, \qquad \mathit{\Gamma}^3 = \mathcal{C}_2^z = \begin{pmatrix} -1 & 0 & 0 \\ 0 & -1 & 0 \\ 0 & 0 & 1 \end{pmatrix}, \tag{S17}$$

$$\mathit{\Gamma}^4 = \mathcal{P}\mathcal{C}_2^y = \begin{pmatrix} 1 & 0 & 0 \\ 0 & -1 & 0 \\ 0 & 0 & 1 \end{pmatrix}, \qquad \mathit{\Gamma}^5 = \mathcal{P}\mathcal{C}_2^z = \begin{pmatrix} 1 & 0 & 0 \\ 0 & 1 & 0 \\ 0 & 0 & -1 \end{pmatrix}, \qquad \mathit{\Gamma}^6 = \mathcal{C}_3^{1z} = \begin{pmatrix} -\frac{1}{2} & \frac{\sqrt{3}}{2} & 0 \\ -\frac{\sqrt{3}}{2} & -\frac{1}{2} & 0 \\ 0 & 0 & 1 \end{pmatrix}, \tag{S18}$$

$$\mathit{\Gamma}^7 = \mathcal{C}_4^{-1z} = \begin{pmatrix} 0 & 1 & 0 \\ -1 & 0 & 0 \\ 0 & 0 & 1 \end{pmatrix}, \qquad \mathit{\Gamma}^8 = \mathcal{P}\mathcal{C}_4^{-1z} = \begin{pmatrix} 0 & -1 & 0 \\ 1 & 0 & 0 \\ 0 & 0 & -1 \end{pmatrix}, \quad \mathit{\Gamma}^9 = \begin{pmatrix} 0 & 1 & 0 \\ 0 & 0 & 1 \\ 1 & 0 & 0 \end{pmatrix}, \tag{S19}$$

$$\mathit{\Gamma}^0 = \hat{I} = \begin{pmatrix} 1 & 0 & 0 \\ 0 & 1 & 0 \\ 0 & 0 & 1 \end{pmatrix}, \tag{S20}$$

$$\underline{\mathit{\Gamma}^k} = \mathit{\Gamma}^k \mathcal{T} : k = 0, 1, 2, 3..., 9, \tag{S21}$$

where the $\mathcal{T}$ is the time-reversal operation. In this way, we can obtain the general matrices representations of physical quantities. The general matrices of the Class I and Class III can be found in Table S2.

TABLE S1. The all 3d magenitic point groups.

Class I		Class II	Class III	Class I		Class II	Class III
Schoenfies	International			Schoenfies	International		
C_1	1	$11'$		S_6	$\bar{3}$	$\bar{3}1'$	$\bar{3}'$
S_2	$\bar{1}$	$\bar{1}1'$	$\bar{1}'$	D_3	32	$321'$	$32'$
C_2	2	$21'$	$2'$	C_{3v}	3m	$3\mathrm{m}1'$	$3\mathrm{m}'$
C_{1v}	m	$\mathrm{m}1'$	m'	D_{3d}	$\bar{3}\mathrm{m}$	$\bar{3}\mathrm{m}1'$	$\bar{3}'\mathrm{m}', \bar{3}'\mathrm{m}, \bar{3}\mathrm{m}'$
C_{2h}	2/m	$2/\mathrm{m}1'$	$2/\mathrm{m}', 2'/\mathrm{m}, 2'/\mathrm{m}'$	C_6	6	$61'$	$6'$
D_2	222	$2221'$	$22'2'$	C_{3h}	$\bar{6}$	$\bar{6}1'$	$\bar{6}'$
C_{2v}	2mm	$2\mathrm{mm}1'$	$2\mathrm{m}'\mathrm{m}', 2'\mathrm{mm}'$	C_{6h}	6/m	$6/\mathrm{m}1'$	$6/\mathrm{m}', 6'/\mathrm{m}, 6'/\mathrm{m}'$
D_{2h}	mmm	$\mathrm{mmm}1'$	$\mathrm{m}'\mathrm{m}'\mathrm{m}', \mathrm{mm}'\mathrm{m}', \mathrm{mmm}'$	D_6	622	$6221'$	$62'2', 6'22'$
C_4	4	$41'$	$4'$	C_{6v}	6mm	$6\mathrm{mm}1'$	$6\mathrm{m}'\mathrm{m}', 6'\mathrm{mm}'$
S_4	$\bar{4}$	$\bar{4}1'$	$\bar{4}'$	D_{3h}	$\bar{6}\mathrm{m}2$	$\bar{6}\mathrm{m}21'$	$\bar{6}\mathrm{m}'2', \bar{6}'\mathrm{m}2', \bar{6}'\mathrm{m}'2$
C_{4h}	4/m	$4/\mathrm{m}1'$	$4/\mathrm{m}', 4'/\mathrm{m}, 4'/\mathrm{m}'$				$6'/\mathrm{mmm}', 6'/\mathrm{m}'\mathrm{mm}'$
D_4	422	$4221'$	$42'2', 4'22$	D_{6h}	6/mmm	$6/\mathrm{mmm}1'$	$6/\mathrm{m}'\mathrm{m}'\mathrm{m}', 6/\mathrm{m}'\mathrm{mm}$
C_{4v}	4mm	$4\mathrm{mm}1'$	$4\mathrm{m}'\mathrm{m}', 4'\mathrm{mm}'$				$6/\mathrm{mm}'\mathrm{m}'$
D_{2d}	$\bar{4}2\mathrm{m}$	$\bar{4}2\mathrm{m}1'$	$\bar{4}'2\mathrm{m}', \bar{4}'2'\mathrm{m}, \bar{4}2'\mathrm{m}'$	T	23	$231'$	
			$4/\mathrm{m}'\mathrm{m}'\mathrm{m}', 4/\mathrm{m}'\mathrm{mm},$	T_h	m3	$\mathrm{m}31'$	$\mathrm{m}'3$
D_{4h}	4/mmm	$4/\mathrm{mmm}1'$	$4'/\mathrm{mmm}', 4'/\mathrm{m}'\mathrm{mm}'$	O	432	$4321'$	$4'32$
			$4/\mathrm{mm}'\mathrm{m}'$	T_d	$\bar{4}3\mathrm{m}$	$\bar{4}3\mathrm{m}1'$	$\bar{4}'3\mathrm{m}'$
C_3	3	$31'$		O_h	m3m	$\mathrm{m}3\mathrm{m}1'$	$\mathrm{m}'3\mathrm{m}', \mathrm{m}'3\mathrm{m}, \mathrm{m}3\mathrm{m}'$

TABLE S2. Generation matrices of MPGs of the Class I and III.

MPG	Generation matrix	MPG	Generation matrix	MPG	Generation matrix	MPG	Generation matrix
1	Γ^0	$\bar{4}'$	$\Gamma^0, \Gamma^3, \underline{\Gamma^8}$	$\bar{3}'$	$\Gamma^0, \Gamma^6, \underline{\Gamma^1}$	$\bar{6}$m2	$\Gamma^0, \Gamma^2, \Gamma^5, \Gamma^6$
$\bar{1}$	Γ^0, Γ^1	4/m	$\Gamma^0, \Gamma^1, \Gamma^7$	32	$\Gamma^0, \Gamma^2, \Gamma^6$	$\bar{6}$m′2′	$\Gamma^0, \Gamma^5, \Gamma^6, \underline{\Gamma^2}$
$\bar{1}'$	$\Gamma^0, \underline{\Gamma^1}$	4/m′	$\Gamma^0, \Gamma^7, \underline{\Gamma^1}$	32′	$\Gamma^0, \Gamma^6, \underline{\Gamma^2}$	$\bar{6}'$m2′	$\Gamma^0, \Gamma^4, \Gamma^6, \underline{\Gamma^2}$
2	Γ^0, Γ^3	4′/m	$\Gamma^0, \Gamma^1, \Gamma^3, \underline{\Gamma^7}$	3m	$\Gamma^0, \Gamma^4, \Gamma^6$	$\bar{6}'$m′2	$\Gamma^0, \Gamma^2, \Gamma^6, \underline{\Gamma^5}$
2′	$\Gamma^0, \underline{\Gamma^3}$	4′/m′	$\Gamma^0, \Gamma^8, \underline{\Gamma^1}$	3m'	$\Gamma^0, \Gamma^6, \underline{\Gamma^4}$	6/mmm	$\Gamma^0, \Gamma^1, \Gamma^2, \Gamma^3, \Gamma^6$
m	Γ^0, Γ^5	422	$\Gamma^0, \Gamma^2, \Gamma^7$	$\bar{3}$m	$\Gamma^0, \Gamma^1, \Gamma^2, \Gamma^6$	6′/mmm′	$\Gamma^0, \Gamma^2, \Gamma^5, \Gamma^6, \underline{\Gamma^1}$
m′	$\Gamma^0, \underline{\Gamma^5}$	42′2′	$\Gamma^0, \Gamma^7, \underline{\Gamma^2}$	$\bar{3}'$m′	$\Gamma^0, \Gamma^2, \Gamma^6, \underline{\Gamma^1}$	6′/m′mm′	$\Gamma^0, \Gamma^1, \Gamma^2, \Gamma^6, \underline{\Gamma^2}$
2/m	$\Gamma^0, \Gamma^1, \Gamma^3$	4′22	$\Gamma^0, \Gamma^2, \Gamma^3, \underline{\Gamma^7}$	$\bar{3}'m$	$\Gamma^0, \Gamma^4, \Gamma^6, \underline{\Gamma^1}$	6/m′m′m′	$\Gamma^0, \Gamma^2, \Gamma^3, \Gamma^6, \underline{\Gamma^1}$
2/m′	$\Gamma^0, \Gamma^3, \underline{\Gamma^1}$	4mm	$\Gamma^0, \Gamma^4, \Gamma^7$	$\bar{3}m'$	$\Gamma^0, \Gamma^1, \Gamma^6, \underline{\Gamma^2}$	6/m′mm	$\Gamma^0, \Gamma^3, \Gamma^4, \Gamma^6, \underline{\Gamma^1}$
2′/m	$\Gamma^0, \Gamma^5, \underline{\Gamma^1}$	4m′m′	$\Gamma^0, \Gamma^7, \underline{\Gamma^4}$	6	$\Gamma^0, \Gamma^3, \Gamma^6$	6/mm′m′	$\Gamma^0, \Gamma^1, \Gamma^3, \Gamma^6, \underline{\Gamma^2}$
2′/m′	$\Gamma^0, \Gamma^1, \underline{\Gamma^3}$	4′mm′	$\Gamma^0, \Gamma^3, \Gamma^4, \underline{\Gamma^7}$	6′	$\Gamma^0, \Gamma^6, \underline{\Gamma^3}$	23	$\Gamma^0, \Gamma^3, \Gamma^9$
222	$\Gamma^0, \Gamma^2, \Gamma^3$	$\bar{4}$2m	$\Gamma^0, \Gamma^2, \Gamma^8$	$\bar{6}$	$\Gamma^0, \Gamma^5, \Gamma^6$	m3	$\Gamma^0, \Gamma^1, \Gamma^3, \Gamma^9$
22′2′	$\Gamma^0, \Gamma^3, \underline{\Gamma^2}$	$\bar{4}$2′m′	$\Gamma^0, \Gamma^8, \underline{\Gamma^4}$	$\bar{6}'$	$\Gamma^0, \Gamma^6, \underline{\Gamma^5}$	m′3	$\Gamma^0, \Gamma^3, \Gamma^9, \underline{\Gamma^1}$
2mm	$\Gamma^0, \Gamma^3, \Gamma^4$	$\bar{4}'$2m′	$\Gamma^0, \Gamma^2, \Gamma^3, \underline{\Gamma^4}$	6/m	$\Gamma^0, \Gamma^1, \Gamma^3, \Gamma^6$	432	$\Gamma^0, \Gamma^7, \Gamma^9$
2m′m′	$\Gamma^0, \Gamma^3, \underline{\Gamma^4}$	$\bar{4}'$2′m	$\Gamma^0, \Gamma^3, \Gamma^4, \underline{\Gamma^8}$	6/m′	$\Gamma^0, \Gamma^3, \Gamma^6, \underline{\Gamma^1}$	4′32	$\Gamma^0, \Gamma^3, \Gamma^9, \underline{\Gamma^7}$
2′mm′	$\Gamma^0, \Gamma^4, \underline{\Gamma^3}$	4/mmm	$\Gamma^0, \Gamma^1, \Gamma^2, \Gamma^7$	6′/m	$\Gamma^0, \Gamma^5 . \Gamma^6, \underline{\Gamma^1}$	$\bar{4}$3m	$\Gamma^0, \Gamma^8, \Gamma^9$
mmm	$\Gamma^0, \Gamma^1, \Gamma^2, \Gamma^3$	4/m′m′m′	$\Gamma^0, \Gamma^2, \Gamma^7, \underline{\Gamma^1}$	6′/m′	$\Gamma^0, \Gamma^1, \Gamma^6, \underline{\Gamma^3}$	$\bar{4}'$3m′	$\Gamma^0, \Gamma^3, \Gamma^9, \underline{\Gamma^8}$
mm′m′	$\Gamma^0, \Gamma^1, \Gamma^3, \underline{\Gamma^2}$	4/m′mm	$\Gamma^0, \Gamma^4, \Gamma^7, \underline{\Gamma^1}$	622	$\Gamma^0, \Gamma^2, \Gamma^3, \Gamma^6$	m3m	$\Gamma^0, \Gamma^1, \Gamma^7, \Gamma^9$
m′m′m′	$\Gamma^0, \Gamma^2, \Gamma^3, \underline{\Gamma^1}$	4′/mmm′	$\Gamma^0, \Gamma^1, \Gamma^2, \Gamma^3, \underline{\Gamma^7}$	62′2′	$\Gamma^0, \Gamma^3, \Gamma^6, \underline{\Gamma^2}$	m′3m′	$\Gamma^0, \Gamma^7, \Gamma^9, \underline{\Gamma^1},$
m′mm	$\Gamma^0, \Gamma^3, \Gamma^4, \underline{\Gamma^1}$	4′/m′mm′	$\Gamma^0, \Gamma^2, \Gamma^8, \underline{\Gamma^1}$	6′22′	$\Gamma^0, \Gamma^2, \Gamma^6, \underline{\Gamma^3}$	m′3m	$\Gamma^0, \Gamma^8, \Gamma^9, \underline{\Gamma^1}$
4	Γ^0, Γ^7	4/mm′m′	$\Gamma^0, \Gamma^1, \Gamma^7, \underline{\Gamma^2}$	6mm	$\Gamma^0, \Gamma^3, \Gamma^4, \Gamma^6$	m3m′	$\Gamma^0, \Gamma^1, \Gamma^3, \Gamma^9, \underline{\Gamma^7}$
4′	$\Gamma^0, \Gamma^3, \underline{\Gamma^7}$	3	Γ^0, Γ^6	6m′m′	$\Gamma^0, \Gamma^3, \Gamma^6, \underline{\Gamma^4}$		
$\bar{4}$	Γ^0, Γ^8	$\bar{3}$	$\Gamma^0, \Gamma^1, \Gamma^6$	6′mm′	$\Gamma^0, \Gamma^4, \Gamma^6, \underline{\Gamma^3}$		

SIII. SYMMETRY ANALYSIS

Since there are published papers working on some groups and symmetry analysis, here we give a comparison between their derivation and this work.

TABLE S3. The comparison of symmetry analysis between other derivations and this work.

Research Object	Symmetry Analysis	References
D_{cd}	List the allowed point group: $C_1, C_{1v}, O, T, D_{2d}, S_4, C_n, C_{nv}, D_n (n = 2, 3, 4, 6)$.	Ref. [1]
χ_{abc}^{BCD}	Give the 2d matrix representations for 16 point group: $C_1, C_{1v}, D_{2d}, S_4, C_n, C_{nv}, D_n (n = 2, 3, 4, 6)$.	Ref. [8]
$\chi_{xyy}^{\text{BCD/BCP}}$ and $\chi_{yxx}^{\text{BCD/BCP}}$	List and compare the contraints of common point group operations.	Ref. [4]
$\chi_{abc}^{\text{BCD/BCP/D}}$	Classify the MPGs by the existence or absence $\chi_{abc}^{\text{BCD/BCP/D}}$.	Ref. [3]
$\chi_{abc}^{\text{BCD(s)}}$	List the conditions of the Néel vector in 15 allowed $\mathcal{PT}$-MPGs (except for $\bar{1}'$).	Ref. [9]
$\chi_{abc}^{\text{BCD/BCP/D}}$ and $\chi_{abc}^{\text{BCD/BCP/D(s)}}$	Present the allowed MPGs and all matrix representations.	In this work

A. The second-order charge response tensor

Firstly, we give a list of all the MPGs that allow χ^{BCD} in Table S4. The first column is the class of the MPGs, where the parentheses indicate the number of MPGs allowed and the total number of MPGs. For instance, the second row, the first column tells us that there are 16 of 32 Class I MPGs in which the χ^{BCD} exists. The last column point out the sequence number of the table represented by the corresponding matrix representations. The corresponding results of the BCP and Drude terms are shown in Table S5 and S6.

It is worth to mentioning that the second-order charge response tensor also can be represented as a rank-two pseudo-tensor Berry curvature dipole $D_{ab} = \int_k f_0 (\partial_a \Omega_b)$. According to the Eq. S8, we have

$$\chi_{abc}^{\text{BCD}} = \frac{e^2\tau}{\hbar}\epsilon_{abd} D_{cd} \tag{S22}$$

For the MPG 23 (T) and 432(O), we have

$$D = \begin{pmatrix} D_{xx} & 0 & 0 \\ 0 & D_{xx} & 0 \\ 0 & 0 & D_{xx} \end{pmatrix} \tag{S23}$$

Hence, the second-order charge response tensor can be written,

$$\chi_x^{\text{BCD}} = \begin{pmatrix} 0 & 0 & 0 \\ 0 & 0 & xyz \\ 0 & -xyz & 0 \end{pmatrix} \; \chi_y^{\text{BCD}} = \begin{pmatrix} 0 & 0 & -xyz \\ 0 & 0 & 0 \\ xyz & 0 & 0 \end{pmatrix} \; \chi_z^{\text{BCD}} = \begin{pmatrix} 0 & xyz & 0 \\ -xyz & 0 & 0 \\ 0 & 0 & 0 \end{pmatrix} \tag{S24}$$

Hence we can get the charge current

$$j_x = \sum_{bc} \chi_{xbc}^{\text{BCD}} E_b E_c = \left(\chi_{xyz}^{\text{BCD}} + \chi_{xzy}^{\text{BCD}}\right) E_y E_z = \left(\chi_{xyz}^{\text{BCD}} - \chi_{xyz}^{\text{BCD}}\right) E_y E_z = 0, \tag{S25}$$

$$j_y = \sum_{bc} \chi_{ybc}^{\text{BCD}} E_b E_c = 0, \tag{S26}$$

$$j_z = \sum_{bc} \chi_{zbc}^{\text{BCD}} E_b E_c = 0. \tag{S27}$$

Hence, there is no second-order response current for the MPGs $T(23)$and $O(432)$.

TABLE S4. 53 MPGs classified by the existence of χ^{BCD}.

Class	MPGs	Matrix representations
I (16/32)	1, 2, 3, 4, 6, m, 2mm, 3m, 4mm, 6mm, 222, 32, 422, 622, $\bar{4}$2m, $\bar{4}$	Table S10
II (16/32)	11′, 21′, 31′, 41′, 61′, m1′, 2mm1′, 3m1′, 4mm1′, 6mm1′, 2221′, 321′, 4221′, 6221′, $\bar{4}$2m1′, $\bar{4}$1′	Table S11
III (21/58)	2′, m′, 22′2′, 2m′m′, 2′mm′, 4′, 6′, 4′22, 42′2′, 32′, 6′22′, 62′2′, $\bar{4}$2′m′, $\bar{4}$′2′m, $\bar{4}$′2m′, $\bar{4}$′, 4′mm′ 6′mm′, 4m′m′, 3m′, 6m′m′	Table S11

TABLE S5. 53 MPGs classified by the existence of χ^{BCP}.

Class	MPGs	Matrix representations
I (16/32)	1, 2, 3, 4, 6, m, 2mm, 3m, 4mm, 6mm, 222, 32, 422, 622, $\bar{4}$2m, $\bar{4}$	Table S12
II(0/32)	none	
III(37/58)	$\bar{1}$′, 2′, m′, 2/m′, 2′/m, 22′2′, 2m′m′, 2′mm′, m′m′m′, m′mm, 4′, $\bar{4}$′, 4/m′, 4′/m′, 4′22, 42′2′, 4′mm′, 4m′m′, $\bar{4}$′2′m, $\bar{4}$2′m′, 4/m′mm, 4′/m′mm′, $\bar{4}$′2m′, 4/m′m′m′, 3m′, $\bar{3}$′, 32′, $\bar{3}$′m′, $\bar{3}$′m, $\bar{6}$′, 6/m′, 62′2′, 6m′m′, $\bar{6}$′m′2, $\bar{6}$′m2′, 6/m′mm, 6/m′m′m′	Table S13

TABLE S6. 58 MPGs classified by the existence of χ^{D}.

Class	MPGs	Matrix representations
I(18/32)	1, 2, m, 222, 2mm, 4, $\bar{4}$, 4mm, $\bar{4}$2m, 3, 32, 3m, 6, $\bar{6}$, 6mm, $\bar{6}$m2, 23, $\bar{4}$3m	Table S14
II (0/32)	none	
III(40/58)	$\bar{1}$′, 2′, m′, 2/m′, 2′/m, 22′2′, 2m′m′, 2′mm′, m′m′m′, m′mm, 4′, $\bar{4}$′, 4/m′, 4′/m′, 4′22, 42′2′, 4′mm′, $\bar{4}$′2′m, $\bar{4}$2′m′, 4/m′mm, 4′/m′mm′, $\bar{3}$′, 3m′, 32′, $\bar{3}$′m′, $\bar{3}$′m, 6′, $\bar{6}$′, 6/m′, 6′/m, 6′22′, 62′2′, 6′mm′, $\bar{6}$′m2′, $\bar{6}$m′2′, 6/m′mm, 6′/mmm′, 4′32, m′3m, m′3	Table S15

B. The second-order spin response tensor

In order to distinction, we call these spin-dependent BCD, BCP and Drude terms. The lists of allowed MPGs are shown for spin-dependent BCD (Table S7), BCP (Table S8) and Drude terms (Table S9).

TABLE S7. 37 MPGs classified by the existence of the second-order spin response tensor $\chi^{\mathrm{BCD}(s)}$.

Class	MPGs	Matrix representations
II (0/32)	none	
III (37/58)	$\bar{1}', 2', \mathrm{m}', 2/\mathrm{m}', 2'/\mathrm{m}, 22'2', 2\mathrm{m}'\mathrm{m}', 2'\mathrm{mm}', \mathrm{m}'\mathrm{m}'\mathrm{m}', \mathrm{m}'\mathrm{mm}, 4', \bar{4}', 4/\mathrm{m}', 4'/\mathrm{m}', 42'2', 4'22,$ $4\mathrm{m}'\mathrm{m}', 4'\mathrm{mm}', \bar{4}2'\mathrm{m}', \bar{4}'2\mathrm{m}', \bar{4}'2'\mathrm{m}, 4/\mathrm{m}'\mathrm{m}'\mathrm{m}', 4/\mathrm{m}'\mathrm{mm}, 4'/\mathrm{m}'\mathrm{mm}', \bar{3}', 32', 3\mathrm{m}', \bar{3}'\mathrm{m}', \bar{3}'\mathrm{m},$ $\bar{6}', 6/\mathrm{m}', 62'2', 6\mathrm{m}'\mathrm{m}', \bar{6}'\mathrm{m}2', \bar{6}'\mathrm{m}'2, 6/\mathrm{m}'\mathrm{m}'\mathrm{m}', 6/\mathrm{m}'\mathrm{mm}$	Table S16

TABLE S8. 37 MPGs classified by the existence of the second-order spin response tensor $\chi^{\mathrm{BCP}(s)}$.

Class	MPGs	Matrix representations
II (16/32)	$11', 21', 31', 41', 61', \mathrm{m}1', 2\mathrm{mm}1', 3\mathrm{m}1', 4\mathrm{mm}1', 6\mathrm{mm}1', 2221', 321', 4221', 6221', \bar{4}2\mathrm{m}1', \bar{4}1'$	Table S17
III (21/58)	$2', \mathrm{m}', 2'2'2, 2\mathrm{m}'\mathrm{m}', 2'\mathrm{mm}', 4', \bar{4}', 42'2', 4'22, 4\mathrm{m}'\mathrm{m}', 4'\mathrm{mm}', \bar{4}2'\mathrm{m}', \bar{4}'2\mathrm{m}', \bar{4}'2'\mathrm{m}, 32', 3\mathrm{m}', 6',$ $62'2', 6'22', 6\mathrm{m}'\mathrm{m}', 6'\mathrm{mm}'$	Table S18

TABLE S9. 39 MPGs classified by the existence of the second-order spin response tensor $\chi^{\mathrm{D}(s)}$.

Class	MPGs	Matrix representations
II (18/32)	$11', 21', \mathrm{m}1', 2221', 2\mathrm{mm}1', 41', \bar{4}1', 4\mathrm{mm}1', \bar{4}2\mathrm{m}1', 31', 321', 3\mathrm{m}1', 61', \bar{6}1', 6\mathrm{mm}1', \bar{6}\mathrm{m}21',$ $231', \bar{4}3\mathrm{m}1'$	Table S19
III (21/58)	$2', \mathrm{m}', 22'2', 2\mathrm{m}'\mathrm{m}', 2'\mathrm{mm}', 4', \bar{4}', 4\mathrm{m}'\mathrm{m}', 4'\mathrm{mm}', \bar{4}2'\mathrm{m}', \bar{4}'2'\mathrm{m}, 32', 3\mathrm{m}', 6', \bar{6}', 6\mathrm{m}'\mathrm{m}', 6'\mathrm{mm}',$ $\bar{6}\mathrm{m}'2', \bar{6}'\mathrm{m}'2, \bar{6}'\mathrm{m}2', \bar{4}'3\mathrm{m}'$	Table S20

According to the Table S4−S9, we can find the Class II omitted in Table S5, S6 and S7 but presented in S4, S8 and S9. This issue is due to the time reversal operator. Class II contains $\mathcal{T}$ as an element to satisfy the time reversal symmetry (TRS). If the tensor does not satisfy the TRS, the Class II MPGs must forbid the corresponding current. Under the time reversal operator, we have

$$\chi_{abc}^{\mathrm{BCD/BCP/D}(\sigma)}(\mathbf{k}) \xrightarrow{\mathcal{T}} \chi_{abc}^{\mathrm{BCD/BCP/D}(\bar{\sigma})}(\text{-}\mathbf{k}). \tag{S28}$$

According to Eq. (S8),

$$\begin{aligned} \chi_{abc}^{\mathrm{BCP/D}(\bar{\sigma})}(\text{-}\mathbf{k}) &= -\chi_{abc}^{\mathrm{BCP/D}(\bar{\sigma})}(\mathbf{k}), \\ \chi_{abc}^{\mathrm{BCD}(\bar{\sigma})}(\text{-}\mathbf{k}) &= \chi_{abc}^{\mathrm{BCD}(\bar{\sigma})}(\mathbf{k}). \end{aligned} \tag{S29}$$

For the second charge current contributed by the BCD,

$$\begin{aligned} \chi_{abc}^{\mathrm{BCD}}(\mathbf{k}) &= \chi_{abc}^{\mathrm{BCD}(\uparrow)}(\mathbf{k}) + \chi_{abc}^{\mathrm{BCD}(\downarrow)}(\mathbf{k}) \\ &\xrightarrow{\mathcal{T}} \chi_{abc}^{\mathrm{BCD}(\downarrow)}(-\mathbf{k}) + \chi_{abc}^{\mathrm{BCD}(\uparrow)}(-\mathbf{k}) = \chi_{abc}^{\mathrm{BCD}(\downarrow)}(\mathbf{k}) + \chi_{abc}^{\mathrm{BCD}(\uparrow)}(\mathbf{k}) = \chi_{abc}^{\mathrm{BCD}}(\mathbf{k}). \end{aligned} \tag{S30}$$

Thus, the BCD term satisfies the time reversal symmetry.

For the BCP and Drude terms,

$$\begin{aligned}\chi_{abc}^{\mathrm{BCP/D}}(\mathbf{k}) &= \chi_{abc}^{\mathrm{BCP/D}(\uparrow)}(\mathbf{k}) + \chi_{abc}^{\mathrm{BCP/D}(\downarrow)}(\mathbf{k}) \\ &\xrightarrow{\mathcal{T}} \chi_{abc}^{\mathrm{BCP/D}(\downarrow)}(-\mathbf{k}) + \chi_{abc}^{\mathrm{BCP/D}(\uparrow)}(-\mathbf{k}) = -\chi_{abc}^{\mathrm{BCP/D}(\downarrow)}(\mathbf{k}) - \chi_{abc}^{\mathrm{BCP/D}(\uparrow)}(\mathbf{k}) = -\chi_{abc}^{\mathrm{BCP/D}}(\mathbf{k}),\end{aligned} \tag{S31}$$

they are both zero for the time reversal symmetry. Therefore, the existence of time reversal symmetry enables the BCP and Drude vanishing but BCD existing, which leads to the Class II ($\mathcal{T}$ is an element for Class II MPGs) omitted in Table S5 (BCP) and S6 (Drude) while presented in S4 (BCD).

For the spin current, the spin BCD, BCP and Drude terms are defined as

$$\chi_{abc}^{\mathrm{BCD/BCP/D}(s)}(\mathbf{k}) = \chi_{abc}^{\mathrm{BCD/BCP/D}(\uparrow)}(\mathbf{k}) - \chi_{abc}^{\mathrm{BCD/BCP/D}(\downarrow)}(\mathbf{k}) \tag{S32}$$

By analogy with Eq. (S30) and Eq. (S31), we have

$$\begin{aligned}\chi_{abc}^{\mathrm{BCD}(s)}(\mathbf{k}) &= \chi_{abc}^{\mathrm{BCD}(\uparrow)}(\mathbf{k}) - \chi_{abc}^{\mathrm{BCD}(\downarrow)}(\mathbf{k}) \\ &\xrightarrow{\mathcal{T}} \chi_{abc}^{\mathrm{BCD}(\downarrow)}(-\mathbf{k}) - \chi_{abc}^{\mathrm{BCD}(\uparrow)}(-\mathbf{k}) = \chi_{abc}^{\mathrm{BCD}(\downarrow)}(\mathbf{k}) - \chi_{abc}^{\mathrm{BCD}(\uparrow)}(\mathbf{k}) = -\chi_{abc}^{\mathrm{BCD(s)}}(\mathbf{k}).\end{aligned} \tag{S33}$$

And,

$$\begin{aligned}\chi_{abc}^{\mathrm{BCP/D}(s)}(\mathbf{k}) &= \chi_{abc}^{\mathrm{BCP/D}(\uparrow)}(\mathbf{k}) - \chi_{abc}^{\mathrm{BCP/D}(\downarrow)}(\mathbf{k}) \\ &\xrightarrow{\mathcal{T}} \chi_{abc}^{\mathrm{BCP/D}(\downarrow)}(-\mathbf{k}) - \chi_{abc}^{\mathrm{BCP/D}(\uparrow)}(-\mathbf{k}) = -\chi_{abc}^{\mathrm{BCP/D}(\downarrow)}(\mathbf{k}) + \chi_{abc}^{\mathrm{BCP/D}(\uparrow)}(\mathbf{k}) = \chi_{abc}^{\mathrm{BCP/D}(s)}(\mathbf{k}).\end{aligned} \tag{S34}$$

For time reversal symmetry, the spin-dependent BCD is zero, while the spin-dependent BCP and Drude are allowed. So the Class II omitted in Table S7 (spin-dependent BCD) but presented in Table S8 (spin-dependent BCP) and S9 (spin-dependent Drude).

Another important message is that the third line in Table S4 (BCD for Class III) is identical to the third line in Table S8 (spin-BCP for class III), while the third line in Table S5 (BCP for Class III) is identical to the second line in Table S7 (spin-BCD for class III).

For the second charge conductivity,

$$\begin{aligned}\chi_{abc}^{\mathrm{BCD}} &= \chi_{abc}^{\mathrm{BCD}(\uparrow)} + \chi_{abc}^{\mathrm{BCD}(\downarrow)} \xrightarrow{\mathcal{RT}} \sum_{i,j,k} \mathit{\Gamma}_{ai}^{n}\mathit{\Gamma}_{bj}^{n}\mathit{\Gamma}_{ck}^{n}\chi_{ijk}^{\mathrm{BCD}(\downarrow)} + \sum_{i,j,k} \mathit{\Gamma}_{ai}^{n}\mathit{\Gamma}_{bj}^{n}\mathit{\Gamma}_{ck}^{n}\chi_{ijk}^{\mathrm{BCD}(\uparrow)} \\ \chi_{abc}^{\mathrm{BCP/D}} &= \chi_{abc}^{\mathrm{BCP/D}(\uparrow)} + \chi_{abc}^{\mathrm{BCP/D}(\downarrow)} \xrightarrow{\mathcal{RT}} -\sum_{i,j,k} \mathit{\Gamma}_{ai}^{n}\mathit{\Gamma}_{bj}^{n}\mathit{\Gamma}_{ck}^{n}\chi_{ijk}^{\mathrm{BCP/D}(\downarrow)} - \sum_{i,j,k} \mathit{\Gamma}_{ai}^{n}\mathit{\Gamma}_{bj}^{n}\mathit{\Gamma}_{ck}^{n}\chi_{ijk}^{\mathrm{BCP/D}(\uparrow)}.\end{aligned} \tag{S35}$$

As the component of charge and spin response tensors is unchanged before and after the symmetry operation, we can find every nonzero component of the tensors. Based on Eq. (S35), the condition of non-zero charge current is

$$\begin{aligned}\chi_{abc}^{\mathrm{BCD}(\uparrow)} &= \sum_{i,j,k} \mathit{\Gamma}_{ai}^{n}\mathit{\Gamma}_{bj}^{n}\mathit{\Gamma}_{ck}^{n}\chi_{ijk}^{\mathrm{BCD}(\uparrow)}, \chi_{abc}^{\mathrm{BCD}(\downarrow)} = \sum_{i,j,k} \mathit{\Gamma}_{ai}^{n}\mathit{\Gamma}_{bj}^{n}\mathit{\Gamma}_{ck}^{n}\chi_{ijk}^{\mathrm{BCP/D}(\downarrow)} \\ \chi_{abc}^{\mathrm{BCP/D}(\uparrow)} &= -\sum_{i,j,k} \mathit{\Gamma}_{ai}^{n}\mathit{\Gamma}_{bj}^{n}\mathit{\Gamma}_{ck}^{n}\chi_{ijk}^{\mathrm{BCP/D}(\uparrow)}, \chi_{abc}^{\mathrm{BCP/D}(\downarrow)} = -\sum_{i,j,k} \mathit{\Gamma}_{ai}^{n}\mathit{\Gamma}_{bj}^{n}\mathit{\Gamma}_{ck}^{n}\chi_{ijk}^{\mathrm{BCP/D}(\downarrow)}\end{aligned} \tag{S36}$$

For the second spin conductivity,

$$\begin{aligned}\chi_{abc}^{\mathrm{BCD}(s)} &= \chi_{abc}^{\mathrm{BCD}(\uparrow)} - \chi_{abc}^{\mathrm{BCD}(\downarrow)} \xrightarrow{\mathcal{RT}} \sum_{i,j,k} \mathit{\Gamma}_{ai}^{n}\mathit{\Gamma}_{bj}^{n}\mathit{\Gamma}_{ck}^{n}\chi_{ijk}^{\mathrm{BCD}(\downarrow)} - \sum_{i,j,k} \mathit{\Gamma}_{ai}^{n}\mathit{\Gamma}_{bj}^{n}\mathit{\Gamma}_{ck}^{n}\chi_{ijk}^{\mathrm{BCD}(\uparrow)} \\ \chi_{abc}^{\mathrm{BCP/D}(s)} &= \chi_{abc}^{\mathrm{BCP/D}(\uparrow)} - \chi_{abc}^{\mathrm{BCP/D}(\downarrow)} \xrightarrow{\mathcal{RT}} -\sum_{i,j,k} \mathit{\Gamma}_{ai}^{n}\mathit{\Gamma}_{bj}^{n}\mathit{\Gamma}_{ck}^{n}\chi_{ijk}^{\mathrm{BCP/D}(\downarrow)} + \sum_{i,j,k} \mathit{\Gamma}_{ai}^{n}\mathit{\Gamma}_{bj}^{n}\mathit{\Gamma}_{ck}^{n}\chi_{ijk}^{\mathrm{BCP/D}(\uparrow)}.\end{aligned} \tag{S37}$$

And,

$$\begin{aligned}\chi_{abc}^{\mathrm{BCD}(\uparrow)} &= -\sum_{i,j,k} \mathit{\Gamma}_{ai}^{n}\mathit{\Gamma}_{bj}^{n}\mathit{\Gamma}_{ck}^{n}\chi_{ijk}^{\mathrm{BCD}(\uparrow)}, \chi_{abc}^{\mathrm{BCD}(\downarrow)} = -\sum_{i,j,k} \mathit{\Gamma}_{ai}^{n}\mathit{\Gamma}_{bj}^{n}\mathit{\Gamma}_{ck}^{n}\chi_{ijk}^{\mathrm{BCP/D}(\downarrow)} \\ \chi_{abc}^{\mathrm{BCP/D}(\uparrow)} &= \sum_{i,j,k} \mathit{\Gamma}_{ai}^{n}\mathit{\Gamma}_{bj}^{n}\mathit{\Gamma}_{ck}^{n}\chi_{ijk}^{\mathrm{BCP/D}(\uparrow)}, \chi_{abc}^{\mathrm{BCP/D}(\downarrow)} = \sum_{i,j,k} \mathit{\Gamma}_{ai}^{n}\mathit{\Gamma}_{bj}^{n}\mathit{\Gamma}_{ck}^{n}\chi_{ijk}^{\mathrm{BCP/D}(\downarrow)}\end{aligned} \tag{S38}$$

Because the BCD and spin-dependent BCP have the same independent components, the first line in Eq. (S36) for BCD is the same as the second line in Eq. (S38) for the spin-dependent BCP term. Thus, the third line in Table S4 (BCD for Class III) is identical to the third line in table S8 (spin-BCP for class III).

Since the second line in Eq. (S36) for BCP is the same as the first in Eq. (S37) for spin-dependent BCD term, the third line in Table S5 (BCP for Class III) is identical to the second line in Table S7 (spin-BCD for class III).

However, the Drude term IS interchangeably symmetric for any two indices, and has 10 independent components, which causes the Drude term is special and have more allowed Class III MPGs (Table S6 and S9).

Next we give all corresponding matrix representations and candidate materials in Table S10 − S20.

TABLE S10: Matrix representations and candidate materials of second-order charge response tensor χ^{BCD} for Class I MPGs.

Class I MPGs	σ_x^{BCD}	σ_y^{BCD}	σ_z^{BCD}	Shortened form	Candidate materials
1	$\begin{pmatrix} 0 & 0 & 0 \\ xyx & xyy & xyz \\ xzx & xzy & xzz \end{pmatrix}$	$\begin{pmatrix} -xyx & -xyy & -xyz \\ 0 & 0 & 0 \\ yzx & yzy & yzz \end{pmatrix}$	$\begin{pmatrix} -xzx & -xzy & -xzz \\ -yzx & -yzy & -yzz \\ 0 & 0 & 0 \end{pmatrix}$	A^{BCD}	twisted bilayer graphene [10]*
2	$\begin{pmatrix} 0 & 0 & 0 \\ 0 & 0 & xyz \\ xzx & xzy & 0 \end{pmatrix}$	$\begin{pmatrix} 0 & 0 & -xyz \\ 0 & 0 & 0 \\ yzx & yzy & 0 \end{pmatrix}$	$\begin{pmatrix} -xzx & -xzy & 0 \\ -yzx & -yzy & 0 \\ 0 & 0 & 0 \end{pmatrix}$	B^{BCD}	$LiFeP_2O_7$ [11]
m	$\begin{pmatrix} 0 & 0 & 0 \\ xyx & xyy & 0 \\ 0 & 0 & xzz \end{pmatrix}$	$\begin{pmatrix} -xyx & -xyy & 0 \\ 0 & 0 & 0 \\ 0 & 0 & yzz \end{pmatrix}$	$\begin{pmatrix} 0 & 0 & -xzz \\ 0 & 0 & -yzz \\ 0 & 0 & 0 \end{pmatrix}$	C^{BCD}	Bilayer WTe_2 [12]*, Graphene [13]*
222	$\begin{pmatrix} 0 & 0 & 0 \\ 0 & 0 & xyz \\ 0 & xzy & 0 \end{pmatrix}$	$\begin{pmatrix} 0 & 0 & -xyz \\ 0 & 0 & 0 \\ yzx & 0 & 0 \end{pmatrix}$	$\begin{pmatrix} 0 & -xzy & 0 \\ -yzx & 0 & 0 \\ 0 & 0 & 0 \end{pmatrix}$	D^{BCD}	$FePO_4$ [14]
2mm	$\begin{pmatrix} 0 & 0 & 0 \\ 0 & 0 & 0 \\ xzx & 0 & 0 \end{pmatrix}$	$\begin{pmatrix} 0 & 0 & 0 \\ 0 & 0 & 0 \\ 0 & yzy & 0 \end{pmatrix}$	$\begin{pmatrix} -xzx & 0 & 0 \\ 0 & -yzy & 0 \\ 0 & 0 & 0 \end{pmatrix}$	E^{BCD}	$FeSb_2O_4$ [15]
4<br>3<br>6	$\begin{pmatrix} 0 & 0 & 0 \\ 0 & 0 & xyz \\ xzx & xzy & 0 \end{pmatrix}$	$\begin{pmatrix} 0 & 0 & -xyz \\ 0 & 0 & 0 \\ -xzy & xzx & 0 \end{pmatrix}$	$\begin{pmatrix} -xzx & -xzy & 0 \\ xzy & -xzx & 0 \\ 0 & 0 & 0 \end{pmatrix}$	F^{BCD}	Ce_5TeO_8 [16]<br>Cu_2OSeO_3 [17]<br>$ScMnO_3$ [18]
$\bar{4}$	$\begin{pmatrix} 0 & 0 & 0 \\ 0 & 0 & 0 \\ xzx & xzy & 0 \end{pmatrix}$	$\begin{pmatrix} 0 & 0 & 0 \\ 0 & 0 & 0 \\ xzy & -xzx & 0 \end{pmatrix}$	$\begin{pmatrix} -xzx & -xzy & 0 \\ -xzy & xzx & 0 \\ 0 & 0 & 0 \end{pmatrix}$	H^{BCD}	–
422<br>32<br>622	$\begin{pmatrix} 0 & 0 & 0 \\ 0 & 0 & xyz \\ 0 & xzy & 0 \end{pmatrix}$	$\begin{pmatrix} 0 & 0 & -xyz \\ 0 & 0 & 0 \\ -xzy & 0 & 0 \end{pmatrix}$	$\begin{pmatrix} 0 & -xzy & 0 \\ xzy & 0 & 0 \\ 0 & 0 & 0 \end{pmatrix}$	I^{BCD}	$Ho_2Ge_2O_7$ [19]<br>$La1/3Sr2/3FeO_3$ [20]<br>–
4mm<br>3m<br>6mm	$\begin{pmatrix} 0 & 0 & 0 \\ 0 & 0 & 0 \\ xzx & 0 & 0 \end{pmatrix}$	$\begin{pmatrix} 0 & 0 & 0 \\ 0 & 0 & 0 \\ 0 & xzx & 0 \end{pmatrix}$	$\begin{pmatrix} -xzx & 0 & 0 \\ 0 & -xzx & 0 \\ 0 & 0 & 0 \end{pmatrix}$	J^{BCD}	–<br>$PbNiO_3$ [21]<br>$(Y,Ho)MnO_3$ [18, 22]
$\bar{4}$2m	$\begin{pmatrix} 0 & 0 & 0 \\ 0 & 0 & 0 \\ 0 & xzy & 0 \end{pmatrix}$	$\begin{pmatrix} 0 & 0 & 0 \\ 0 & 0 & 0 \\ xzy & 0 & 0 \end{pmatrix}$	$\begin{pmatrix} 0 & -xzy & 0 \\ -xzy & 0 & 0 \\ 0 & 0 & 0 \end{pmatrix}$	K^{BCD}	HgTe [23]*

*Nonlinear charge current contributed by the BCD has been reported.

TABLE S11: Matrix representations of second-order charge response tensor χ^{BCD} for Class II and III MPGs.

Class MPGs	III	Shortened form	Candidate materials
$11'$		A^{BCD}	$NiPS_3$ [24]
$21'$<br>$2'$		B^{BCD}	$HoNiO_3$ [11]<br>$U_3Al_2Si_3$ [25]
$m1'$<br>m'		C^{BCD}	Na_2MnF_5 [26]<br>$MnTiO_3$ [27]
$2221'$<br>$22'2'$		D^{BCD}	$AgNiO_2$ [28]<br>(Cs,Rb)$NiCl_3$ [29, 30]
$2mm1'$<br>$2m'm'$<br>$2'mm'$		E^{BCD}	MnS_2 [31]<br>$MnSO_4$ [32], $TbCrO_3$ [33]<br>$MnSe_2$ [34]
$41'$<br>$31'$<br>$61'$<br>$4'$<br>$6'$		F^{BCD}	$CeAuAl_3$ [35]<br>Ni_3TeO_6 [36]<br>–<br>–<br>$YMnO_3$ [37]
$\bar{4}1'$<br>$\bar{4}'$		H^{BCD}	–<br>$CsCoF_4$ [38]
$4221'$<br>$321'$<br>$6221'$<br>$4'22$<br>$42'2'$<br>$32'$<br>$6'22'$<br>$62'2'$		I^{BCD}	MnO_2 [39]<br>$SrFeO_3$ [40]<br>$ScMn_6Ge_6$ [41]<br>$Er_2Ge_2O_7$ [42]<br>Nd_5Si_4 [25]<br>$NaMnFeF_6$ [43]<br>–<br>$EuIn_2As_2$ [44], $DyMn_6Ge_6$ [45]
$4mm1'$<br>$3m1'$<br>$6mm1'$<br>$4'mm'$<br>$4m'm'$<br>$3m'$<br>$6'mm'$<br>$6m'm'$		J^{BCD}	CeAlGe [46]<br>CuMnSb [47]<br>–<br>–<br>Ce(Co,Ir)Ge_3 [48, 49]<br>GaV_4S_8 [50],GaV_4S_8 [50]<br>(Yb,Ho)MnO_3 [37, 51]<br>(Yb,Ho,Sc)MnO_3 [18, 37, 51]
$\bar{4}2m1'$<br>$\bar{4}'2m'$<br>$\bar{4}'2'm$<br>$\bar{4}2'm'$		K^{BCD}	$GeCu_2O_4$ [52]<br>Pb_2MnO_4 [53], Ce_4Sb_3 [54]<br>–<br>$EuCr_2As_2$ [55], $MgCr_2O_4$ [56]

TABLE S12: Matrix representations and candidate materials of second-order charge response tensor χ^{BCP} for Class I MPGs.

Class I MPGs	χ_x^{BCP}	χ_y^{BCP}	χ_z^{BCP}	Shortened form	Candidate materials
1	$\begin{pmatrix} 0 & 0 & 0 \\ xyx & xyy & xyz \\ xzx & xzy & xzz \end{pmatrix}$	$\begin{pmatrix} -xyx & -xyy & -xyz \\ 0 & 0 & 0 \\ yzx & yzy & yzz \end{pmatrix}$	$\begin{pmatrix} -xzx & -xzy & -xzz \\ -yzx & -yzy & -yzz \\ 0 & 0 & 0 \end{pmatrix}$	A^{BCP}	TABEL S11[1]
2	$\begin{pmatrix} 0 & 0 & 0 \\ 0 & 0 & xyz \\ xzx & xzy & 0 \end{pmatrix}$	$\begin{pmatrix} 0 & 0 & -xyz \\ 0 & 0 & 0 \\ yzx & yzy & 0 \end{pmatrix}$	$\begin{pmatrix} -xzx & -xzy & 0 \\ -yzx & -yzy & 0 \\ 0 & 0 & 0 \end{pmatrix}$	B^{BCP}	
m	$\begin{pmatrix} 0 & 0 & 0 \\ xyx & xyy & 0 \\ 0 & 0 & xzz \end{pmatrix}$	$\begin{pmatrix} -xyx & -xyy & 0 \\ 0 & 0 & 0 \\ 0 & 0 & yzz \end{pmatrix}$	$\begin{pmatrix} 0 & 0 & -xzz \\ 0 & 0 & -yzz \\ 0 & 0 & 0 \end{pmatrix}$	C^{BCP}	
222	$\begin{pmatrix} 0 & 0 & 0 \\ 0 & 0 & xyz \\ 0 & xzy & 0 \end{pmatrix}$	$\begin{pmatrix} 0 & 0 & -xyz \\ 0 & 0 & 0 \\ yzx & 0 & 0 \end{pmatrix}$	$\begin{pmatrix} 0 & -xzy & 0 \\ -yzx & 0 & 0 \\ 0 & 0 & 0 \end{pmatrix}$	D^{BCP}	
2mm	$\begin{pmatrix} 0 & 0 & 0 \\ 0 & 0 & 0 \\ xzx & 0 & 0 \end{pmatrix}$	$\begin{pmatrix} 0 & 0 & 0 \\ 0 & 0 & 0 \\ 0 & yzy & 0 \end{pmatrix}$	$\begin{pmatrix} -xzx & 0 & 0 \\ 0 & -yzy & 0 \\ 0 & 0 & 0 \end{pmatrix}$	E^{BCP}	
$4, 3, 6$	$\begin{pmatrix} 0 & 0 & 0 \\ 0 & 0 & xyz \\ xzx & xzy & 0 \end{pmatrix}$	$\begin{pmatrix} 0 & 0 & -xyz \\ 0 & 0 & 0 \\ -xzy & xzx & 0 \end{pmatrix}$	$\begin{pmatrix} -xzx & -xzy & 0 \\ xzy & -xzx & 0 \\ 0 & 0 & 0 \end{pmatrix}$	F^{BCP}	
$\bar{4}$	$\begin{pmatrix} 0 & 0 & 0 \\ 0 & 0 & 0 \\ xzx & xzy & 0 \end{pmatrix}$	$\begin{pmatrix} 0 & 0 & 0 \\ 0 & 0 & 0 \\ xzy & -xzx & 0 \end{pmatrix}$	$\begin{pmatrix} -xzx & -xzy & 0 \\ -xzy & xzx & 0 \\ 0 & 0 & 0 \end{pmatrix}$	H^{BCP}	
$422, 32, 622$	$\begin{pmatrix} 0 & 0 & 0 \\ 0 & 0 & xyz \\ 0 & xzy & 0 \end{pmatrix}$	$\begin{pmatrix} 0 & 0 & -xyz \\ 0 & 0 & 0 \\ -xzy & 0 & 0 \end{pmatrix}$	$\begin{pmatrix} 0 & -xzy & 0 \\ xzy & 0 & 0 \\ 0 & 0 & 0 \end{pmatrix}$	I^{BCP}	
$4\mathrm{mm}, 3\mathrm{m}, 6\mathrm{mm}$	$\begin{pmatrix} 0 & 0 & 0 \\ 0 & 0 & 0 \\ xzx & 0 & 0 \end{pmatrix}$	$\begin{pmatrix} 0 & 0 & 0 \\ 0 & 0 & 0 \\ 0 & xzx & 0 \end{pmatrix}$	$\begin{pmatrix} -xzx & 0 & 0 \\ 0 & -xzx & 0 \\ 0 & 0 & 0 \end{pmatrix}$	J^{BCP}	
$\bar{4}2m$	$\begin{pmatrix} 0 & 0 & 0 \\ 0 & 0 & 0 \\ 0 & xzy & 0 \end{pmatrix}$	$\begin{pmatrix} 0 & 0 & 0 \\ 0 & 0 & 0 \\ xyz & 0 & 0 \end{pmatrix}$	$\begin{pmatrix} 0 & -xzy & 0 \\ -xzy & 0 & 0 \\ 0 & 0 & 0 \end{pmatrix}$	K^{BCP}	

[1] The first column MPGs are same as in TABLE S11, so the candidates materials are not repeated here.

TABLE S13: Matrix representations and candidate materials of second-order charge response tensor χ^{BCP} for Class III MPGs.

Class III MPGs	Shortened form	Candidate materials
$\bar{1}'$	A^{BCP}	Cr_2S_3 [57]
m'<br>$2/m'$	B^{BCP}	$MnTiO_3$ [27]<br>$ErGe_3$ [58]
$2'$<br>$2'/m$	C^{BCP}	$U_3Al_2Si_3$ [25]<br>CuMnAs [3]*, Cr_2O_3 [59]
$2m'm'$<br>$m'm'm'$	D^{BCP}	$MnSO_4$ [32], $TbCrO_3$ [33]<br>$RbFeO_2$ [60]
$22'2', 2'mm'$<br>$m'mm$	E^{BCP}	(Cs,Rb)$NiCl_3$ [29, 30], $MnSe_2$ [34]<br>Mn_2Au [4]*
$\bar{4}'$<br>$4/m'$<br>$\bar{3}'$<br>$\bar{6}'$<br>$6/m'$	F^{BCP}	–<br>NdB_4 [61]<br>Mn(Ti,Ge,Mg)O_3 [62]<br>$Cu_{0.82}Mn_{1.18}As$ [63]<br>$Cu_{14}Au_{51}$ [64]
$4'$<br>$4'/m'$	H^{BCP}	–<br>K(Os,Ru)O_4 [65]
$4m'm', \bar{4}'2m', 3m', 6m'm'$<br>$4/m'm'm'$<br>$\bar{3}'m'$<br>$\bar{6}'m'2$<br>$6/m'm'm'$	I^{BCP}	Ce(Co,Ir)Ge_3 [48, 49], GaV_4S_8 [50],GaV_4S_8 [50], (Yb,Ho,Sc)MnO_3 [18, 37, 51]<br>Bi_2CuO_4 [66]<br>Cr_2O_3 [67]<br>$RbFeCl_3$ [68]<br>–
$42'2', \bar{4}'2'm, 32', 62'2'$<br>$4/m'mm$<br>$\bar{3}'m$<br>$\bar{6}'m2'$<br>$6/m'mm$	J^{BCP}	Nd_5Si_4 [25], $NaMnFeF_6$ [43], $EuIn_2As_2$ [44], $DyMn_6Ge_6$ [45]<br>$Co_3Al_2Si_3O_{12}$ [69]<br>Ca_2(Y,La)$Zr_2Fe_3O_{12}$ [70]<br>$CsFeCl_3$ [71]<br>–
$4'22, 4'mm', \bar{4}2'm'$<br>$4'/m'mm'$	K^{BCP}	$Er_2Ge_2O_7$ [42], $EuCr_2As_2$ [55], $MgCr_2O_4$ [56], $LiFeCr_4O_8$ [72]<br>$CoAl_2O_4$ [73]

*Nonlinear charge current contributed by the BCP has been reported.

TABLE S14: Matrix representations and candidate materials of second-order charge response tensor χ^{D} for Class I MPGs.

Class I MPGs	σ_x^{D}	σ_y^{D}	σ_z^{D}	Shortened form	Candidate materials
1	$\begin{pmatrix} xxx & xxy & xxz \\ xxy & xyy & xyz \\ xxz & xyz & xzz \end{pmatrix}$	$\begin{pmatrix} xxy & xyy & xyz \\ xyy & yyy & yyz \\ xyz & yyz & yzz \end{pmatrix}$	$\begin{pmatrix} xxz & xyz & xzz \\ xyz & yyz & yzz \\ xzz & yzz & zzz \end{pmatrix}$	A^{D}	
2	$\begin{pmatrix} 0 & 0 & xxz \\ 0 & 0 & xyz \\ xxz & xyz & 0 \end{pmatrix}$	$\begin{pmatrix} 0 & 0 & xyz \\ 0 & 0 & yyz \\ xyz & yyz & 0 \end{pmatrix}$	$\begin{pmatrix} xxz & xyz & 0 \\ xyz & yyz & 0 \\ 0 & 0 & zzz \end{pmatrix}$	B^{D}	$LiFeP_2O_7$ [11]
m	$\begin{pmatrix} xxx & xxy & 0 \\ xxy & xyy & 0 \\ 0 & 0 & xzz \end{pmatrix}$	$\begin{pmatrix} xxy & xyy & 0 \\ xyy & yyy & 0 \\ 0 & 0 & yzz \end{pmatrix}$	$\begin{pmatrix} 0 & 0 & xzz \\ 0 & 0 & yzz \\ xzz & yzz & 0 \end{pmatrix}$	C^{D}	$Mn_4Nb_2O_9$ [74]
$222, \bar{4}2m$<br>23<br>$\bar{4}3m$	$\begin{pmatrix} 0 & 0 & 0 \\ 0 & 0 & xyz \\ 0 & xyz & 0 \end{pmatrix}$	$\begin{pmatrix} 0 & 0 & xyz \\ 0 & 0 & 0 \\ xyz & 0 & 0 \end{pmatrix}$	$\begin{pmatrix} 0 & xyz & 0 \\ xyz & 0 & 0 \\ 0 & 0 & 0 \end{pmatrix}$	D^{D}	$FePO_4$ [14], Ce_4Ge_3 [75]<br>$Mn_3Ir(Ge,Si)$ [76, 77]<br>–
2mm	$\begin{pmatrix} 0 & 0 & xxz \\ 0 & 0 & 0 \\ xxz & 0 & 0 \end{pmatrix}$	$\begin{pmatrix} 0 & 0 & 0 \\ 0 & 0 & yyz \\ 0 & yyz & 0 \end{pmatrix}$	$\begin{pmatrix} xxz & 0 & 0 \\ 0 & yyz & 0 \\ 0 & 0 & zzz \end{pmatrix}$	E^{D}	$FeSb_2O_4$ [15]
$4, 4mm, 6, 6mm$	$\begin{pmatrix} 0 & 0 & xxz \\ 0 & 0 & 0 \\ xxz & 0 & 0 \end{pmatrix}$	$\begin{pmatrix} 0 & 0 & 0 \\ 0 & 0 & xxz \\ 0 & xxz & 0 \end{pmatrix}$	$\begin{pmatrix} xxz & 0 & 0 \\ 0 & xxz & 0 \\ 0 & 0 & zzz \end{pmatrix}$	F^{D}	Ce_5TeO_8 [16], $ScMnO_3$ [18], $(Y,Ho)MnO_3$ [18, 22]
$\bar{4}$	$\begin{pmatrix} 0 & 0 & xxz \\ 0 & 0 & xyz \\ xxz & xyz & 0 \end{pmatrix}$	$\begin{pmatrix} 0 & 0 & xyz \\ 0 & 0 & -xxz \\ xyz & -xxz & 0 \end{pmatrix}$	$\begin{pmatrix} xxz & xyz & 0 \\ xyz & -xxz & 0 \\ 0 & 0 & 0 \end{pmatrix}$	H^{D}	–
32<br>$\bar{6}m2$	$\begin{pmatrix} xxx & 0 & 0 \\ 0 & -xxx & 0 \\ 0 & 0 & 0 \end{pmatrix}$	$\begin{pmatrix} 0 & -xxx & 0 \\ -xxx & 0 & 0 \\ 0 & 0 & 0 \end{pmatrix}$	0	I^{D}	$La1/3Sr2/3FeO_3$ [20]<br>$PbTaSe_2$ [78]*
3	$\begin{pmatrix} xxx & -yyy & xxz \\ -yyy & -xxx & 0 \\ xxz & 0 & 0 \end{pmatrix}$	$\begin{pmatrix} -yyy & -xxx & 0 \\ -xxx & yyy & xxz \\ 0 & xxz & 0 \end{pmatrix}$	$\begin{pmatrix} xxz & 0 & 0 \\ 0 & xxz & 0 \\ 0 & 0 & zzz \end{pmatrix}$	J^{D}	Cu_2OSeO_3 [17]
3m	$\begin{pmatrix} 0 & -yyy & xxz \\ -yyy & 0 & 0 \\ xxz & 0 & 0 \end{pmatrix}$	$\begin{pmatrix} -yyy & 0 & 0 \\ 0 & yyy & xxz \\ 0 & xxz & 0 \end{pmatrix}$	$\begin{pmatrix} xxz & 0 & 0 \\ 0 & xxz & 0 \\ 0 & 0 & zzz \end{pmatrix}$	K^{D}	(111)-$LaAlO_3/SrTiO_3$ interface [79]*
$\bar{6}$	$\begin{pmatrix} xxx & -yyy & 0 \\ -yyy & -xxx & 0 \\ 0 & 0 & 0 \end{pmatrix}$	$\begin{pmatrix} -yyy & -xxx & 0 \\ -xxx & yyy & 0 \\ 0 & 0 & 0 \end{pmatrix}$	0	L^{D}	–

*Nonlinear charge current contributed by the Drude has been reported.

TABLE S15: Matrices expressions of second-order charge response tensor χ^{D} for Class III MPGs.

Class III MPGs	shortened form	Candidate materials
$\bar{1}'$	A^{D}	Cr_2S_3 [57]
$\mathrm{m}', 2/\mathrm{m}'$	B^{D}	$MnTiO_3$ [27], $ErGe_3$ [58]
$2', 2'/\mathrm{m}$	C^{D}	$U_3Al_2Si_3$ [25], Cr_2O_3 [59]
$4'32$<br>$\mathrm{m}'3\mathrm{m}$<br>$\mathrm{m}'3$<br>$4'\mathrm{mm}'$<br>$2\mathrm{m}'\mathrm{m}', \mathrm{m}'\mathrm{m}'\mathrm{m}', 4'22$<br>$\bar{4}2'\mathrm{m}', 4'/\mathrm{m}'\mathrm{mm}'$	D^{D}	$BaCuTe_2O_6$ [80]<br>–<br>–<br>–<br>$MnSO_4$ [32], $TbCrO_3$ [33], $RbFeO_2$ [60]<br>$Er_2Ge_2O_7$ [42], $EuCr_2As_2$ [55], $CoAl_2O_4$ [73]
$22'2', 2'\mathrm{mm}', \mathrm{m}'\mathrm{mm}$	E^{D}	(Cs,Rb)$NiCl_3$ [29, 30], $MnSe_2$ [34], Mn_2Au [81]
$\bar{4}', 4/\mathrm{m}', 42'2'$<br>$\bar{4}'2'\mathrm{m}, 4/\mathrm{m}'\mathrm{mm}, \bar{6}'$<br>$6/\mathrm{m}', 62'2', \bar{6}'\mathrm{m}2'$<br>$6/\mathrm{m}'\mathrm{mm}$	F^{D}	NdB_4 [61], Nd_5Si_4 [25]<br>$Co_3Al_2Si_3O_{12}$ [69], $Cu_{0.82}Mn_{1.18}As$ [63]<br>$Cu_{14}Au_{51}$ [64], $EuIn_2As_2$ [44], $DyMn_6Ge_6$ [45], $CsFeCl_3$ [71]<br>–
$4', 4'/\mathrm{m}'$	H^{D}	K(Os,Ru)O_4 [65]
$\bar{3}'$	I^{D}	Mn(Ti,Ge,Mg)O_3 [62]
$\bar{6}\mathrm{m}'2'$<br>$6'/\mathrm{mmm}'$<br>$3\mathrm{m}', \bar{3}'\mathrm{m}', 6'22', 6'\mathrm{mm}'$	J^{D}	$CsMnBr_3$ [82]<br>–<br>GaV_4S_8 [50],GaV_4S_8 [50], Cr_2O_3 [67], (Yb,Ho)MnO_3 [37, 51]
$32', \bar{3}'\mathrm{m}$	K^{D}	$NaMnFeF_6$ [43], Ca_2(Y,La)$Zr_2Fe_3O_{12}$ [70]
$6'$<br>$6'/\mathrm{m}$	L^{D}	$YMnO_3$ [37]<br>$U_{14}Au_{51}$ [83]

TABLE S16: Matrix representations of second-order spin response tensor $\chi^{\mathrm{BCD(s)}}$ for Class III MPGs.

Class III MPGs	$\chi_x^{\mathrm{BCD(s)}}$	$\chi_y^{\mathrm{BCD(s)}}$	$\chi_z^{\mathrm{BCD(s)}}$	Shortened Form
$\bar{1}'$	$\begin{pmatrix} 0 & 0 & 0 \\ xyx & xyy & xyz \\ xzx & xzy & xzz \end{pmatrix}$	$\begin{pmatrix} -xyx & -xyy & -xyz \\ 0 & 0 & 0 \\ yzx & yzy & yzz \end{pmatrix}$	$\begin{pmatrix} -xzx & -xzy & -xzz \\ -yzx & -yzy & -yzz \\ 0 & 0 & 0 \end{pmatrix}$	$A^{\mathrm{BCD}(s)}$
$\mathrm{m}', 2/\mathrm{m}'$	$\begin{pmatrix} 0 & 0 & 0 \\ 0 & 0 & xyz \\ xzx & xzy & 0 \end{pmatrix}$	$\begin{pmatrix} 0 & 0 & -xyz \\ 0 & 0 & 0 \\ yzx & yzy & 0 \end{pmatrix}$	$\begin{pmatrix} -xzx & -xzy & 0 \\ -yzx & -yzy & 0 \\ 0 & 0 & 0 \end{pmatrix}$	$B^{\mathrm{BCD}(s)}$
$2', 2'/\mathrm{m}$	$\begin{pmatrix} 0 & 0 & 0 \\ xyx & xyy & 0 \\ 0 & 0 & xzz \end{pmatrix}$	$\begin{pmatrix} -xyx & -xyy & 0 \\ 0 & 0 & 0 \\ 0 & 0 & yzz \end{pmatrix}$	$\begin{pmatrix} 0 & 0 & -xzz \\ 0 & 0 & -yzz \\ 0 & 0 & 0 \end{pmatrix}$	$C^{\mathrm{BCD}(s)}$
$2\mathrm{m}'\mathrm{m}', \bar{4}'2\mathrm{m}', \mathrm{m}'\mathrm{m}'\mathrm{m}'$	$\begin{pmatrix} 0 & 0 & 0 \\ 0 & 0 & xyz \\ 0 & xzy & 0 \end{pmatrix}$	$\begin{pmatrix} 0 & 0 & -xyz \\ 0 & 0 & 0 \\ yzx & 0 & 0 \end{pmatrix}$	$\begin{pmatrix} 0 & -xzy & 0 \\ -yzx & 0 & 0 \\ 0 & 0 & 0 \end{pmatrix}$	$D^{\mathrm{BCD}(s)}$
$22'2, \mathrm{m}'\mathrm{mm}$	$\begin{pmatrix} 0 & 0 & 0 \\ 0 & 0 & 0 \\ xzx & 0 & 0 \end{pmatrix}$	$\begin{pmatrix} 0 & 0 & 0 \\ 0 & 0 & 0 \\ 0 & yzy & 0 \end{pmatrix}$	$\begin{pmatrix} -xzx & 0 & 0 \\ 0 & -yzy & 0 \\ 0 & 0 & 0 \end{pmatrix}$	$E^{\mathrm{BCD}(s)}$
$4/\mathrm{m}', \bar{3}', 6/\mathrm{m}', \bar{4}', \bar{6}'$	$\begin{pmatrix} 0 & 0 & 0 \\ 0 & 0 & xyz \\ xzx & xzy & 0 \end{pmatrix}$	$\begin{pmatrix} 0 & 0 & -xyz \\ 0 & 0 & 0 \\ -xzy & xzx & 0 \end{pmatrix}$	$\begin{pmatrix} zxx & -xzy & 0 \\ xzy & zxx & 0 \\ 0 & 0 & 0 \end{pmatrix}$	$F^{\mathrm{BCD}(s)}$
$4', 4'/\mathrm{m}'$	$\begin{pmatrix} 0 & 0 & 0 \\ 0 & 0 & 0 \\ xzx & xzy & 0 \end{pmatrix}$	$\begin{pmatrix} 0 & 0 & 0 \\ 0 & 0 & 0 \\ xzy & -xzx & 0 \end{pmatrix}$	$\begin{pmatrix} -xzx & -xzy & 0 \\ -xzy & xzx & 0 \\ 0 & 0 & 0 \end{pmatrix}$	$H^{\mathrm{BCD}(s)}$
$4\mathrm{m}'\mathrm{m}', 4/\mathrm{m}'\mathrm{m}'\mathrm{m}', 3\mathrm{m}'$, $\bar{3}'\mathrm{m}', 6\mathrm{m}'\mathrm{m}', 6/\mathrm{m}'\mathrm{m}'\mathrm{m}', \bar{6}'\mathrm{m}'2$	$\begin{pmatrix} 0 & 0 & 0 \\ 0 & 0 & xyz \\ 0 & xzy & 0 \end{pmatrix}$	$\begin{pmatrix} 0 & 0 & -xyz \\ 0 & 0 & 0 \\ -xzy & 0 & 0 \end{pmatrix}$	$\begin{pmatrix} 0 & -xzy & 0 \\ xzy & 0 & 0 \\ 0 & 0 & 0 \end{pmatrix}$	$I^{\mathrm{BCD}(s)}$
$2'\mathrm{mm}'$	$\begin{pmatrix} 0 & 0 & 0 \\ 0 & xyy & 0 \\ 0 & 0 & xzz \end{pmatrix}$	$\begin{pmatrix} 0 & -xyy & 0 \\ 0 & 0 & 0 \\ 0 & 0 & 0 \end{pmatrix}$	$\begin{pmatrix} 0 & 0 & -xzz \\ 0 & 0 & 0 \\ 0 & 0 & 0 \end{pmatrix}$	$L^{\mathrm{BCD}(s)}$
$42'2', 4/\mathrm{m}'\mathrm{mm}$, $32', \bar{3}'\mathrm{m}, 62'2', \bar{6}'\mathrm{m}2', 6/\mathrm{m}'\mathrm{mm}, \bar{4}'2'\mathrm{m}$	$\begin{pmatrix} 0 & 0 & 0 \\ 0 & 0 & 0 \\ xzx & 0 & 0 \end{pmatrix}$	$\begin{pmatrix} 0 & 0 & 0 \\ 0 & 0 & 0 \\ 0 & xzx & 0 \end{pmatrix}$	$\begin{pmatrix} -xzx & 0 & 0 \\ 0 & -xzx & 0 \\ 0 & 0 & 0 \end{pmatrix}$	$M^{\mathrm{BCD}(s)}$
$4'22m, \bar{4}2'\mathrm{m}', 4'/\mathrm{m}'\mathrm{m}'\mathrm{m}$	$\begin{pmatrix} 0 & 0 & 0 \\ 0 & 0 & 0 \\ 0 & xzy & 0 \end{pmatrix}$	$\begin{pmatrix} 0 & 0 & 0 \\ 0 & 0 & 0 \\ xzy & 0 & 0 \end{pmatrix}$	$\begin{pmatrix} 0 & -xzy & 0 \\ -xzy & 0 & 0 \\ 0 & 0 & 0 \end{pmatrix}$	$N^{\mathrm{BCD}(s)}$
$4'\mathrm{mm}'$	$\begin{pmatrix} 0 & 0 & 0 \\ 0 & 0 & 0 \\ xzx & 0 & 0 \end{pmatrix}$	$\begin{pmatrix} 0 & 0 & 0 \\ 0 & 0 & 0 \\ 0 & -xzx & 0 \end{pmatrix}$	$\begin{pmatrix} -xzx & 0 & 0 \\ 0 & xzx & 0 \\ 0 & 0 & 0 \end{pmatrix}$	$O^{\mathrm{BCD}(s)}$

In this table, the candidate materials are not repeated, which are the same as the TABLE S11.

TABLE S17: Matrix representations of second-order spin response tensor $\chi^{\mathrm{BCP(s)}}$ for Class II MPGs.

Class II MPGs	$\chi_x^{\mathrm{BCP(s)}}$	$\chi_y^{\mathrm{BCP(s)}}$	$\chi_z^{\mathrm{BCP(s)}}$	Shortened form
$11'$	$\begin{pmatrix} 0 & 0 & 0 \\ xyx & xyy & xyz \\ xzx & xzy & xzz \end{pmatrix}$	$\begin{pmatrix} -xyx & -xyy & -xyz \\ 0 & 0 & 0 \\ yzx & yzy & yzz \end{pmatrix}$	$\begin{pmatrix} -xzx & -xzy & -xzz \\ -yzx & -yzy & -yzz \\ 0 & 0 & 0 \end{pmatrix}$	$A^{\mathrm{BCP}(s)}$
$21'$	$\begin{pmatrix} 0 & 0 & 0 \\ 0 & 0 & xyz \\ xzx & xzy & 0 \end{pmatrix}$	$\begin{pmatrix} 0 & 0 & -xyz \\ 0 & 0 & 0 \\ yzx & yzy & 0 \end{pmatrix}$	$\begin{pmatrix} -xzx & -xzy & 0 \\ -yzx & -yzy & 0 \\ 0 & 0 & 0 \end{pmatrix}$	$B^{\mathrm{BCP}(s)}$
$\mathrm{m}1'$	$\begin{pmatrix} 0 & 0 & 0 \\ xyx & xyy & 0 \\ 0 & 0 & xzz \end{pmatrix}$	$\begin{pmatrix} -xyx & -xyy & 0 \\ 0 & 0 & 0 \\ 0 & 0 & yzz \end{pmatrix}$	$\begin{pmatrix} 0 & 0 & -xzz \\ 0 & 0 & -yzz \\ 0 & 0 & 0 \end{pmatrix}$	$C^{\mathrm{BCP}(s)}$
$2221'$	$\begin{pmatrix} 0 & 0 & 0 \\ 0 & 0 & xyz \\ 0 & xzy & 0 \end{pmatrix}$	$\begin{pmatrix} 0 & 0 & -xyz \\ 0 & 0 & 0 \\ yzx & 0 & 0 \end{pmatrix}$	$\begin{pmatrix} 0 & -xzy & 0 \\ -yzx & 0 & 0 \\ 0 & 0 & 0 \end{pmatrix}$	$D^{\mathrm{BCP}(s)}$
$2\mathrm{mm}1'$	$\begin{pmatrix} 0 & 0 & 0 \\ 0 & 0 & 0 \\ xzx & 0 & 0 \end{pmatrix}$	$\begin{pmatrix} 0 & 0 & 0 \\ 0 & 0 & 0 \\ 0 & yzy & 0 \end{pmatrix}$	$\begin{pmatrix} -xzx & 0 & 0 \\ 0 & -yzy & 0 \\ 0 & 0 & 0 \end{pmatrix}$	$E^{\mathrm{BCP}(s)}$
$41', 31', 61'$	$\begin{pmatrix} 0 & 0 & 0 \\ 0 & 0 & xyz \\ xzx & xzy & 0 \end{pmatrix}$	$\begin{pmatrix} 0 & 0 & -xyz \\ 0 & 0 & 0 \\ -xzy & xzx & 0 \end{pmatrix}$	$\begin{pmatrix} -xzx & -xzy & 0 \\ xzy & -xzx & 0 \\ 0 & 0 & 0 \end{pmatrix}$	$F^{\mathrm{BCP}(s)}$
$\bar{4}1',$	$\begin{pmatrix} 0 & 0 & 0 \\ 0 & 0 & 0 \\ xzx & xzy & 0 \end{pmatrix}$	$\begin{pmatrix} 0 & 0 & 0 \\ 0 & 0 & 0 \\ xzy & -xzx & 0 \end{pmatrix}$	$\begin{pmatrix} -xzx & -xzy & 0 \\ -xzy & xzx & 0 \\ 0 & 0 & 0 \end{pmatrix}$	$H^{\mathrm{BCP}(s)}$
$4221', 321', 6221'$	$\begin{pmatrix} 0 & 0 & 0 \\ 0 & 0 & xyz \\ 0 & xzy & 0 \end{pmatrix}$	$\begin{pmatrix} 0 & 0 & -xyz \\ 0 & 0 & 0 \\ -xzy & 0 & 0 \end{pmatrix}$	$\begin{pmatrix} 0 & -xzy & 0 \\ xzy & 0 & 0 \\ 0 & 0 & 0 \end{pmatrix}$	$I^{\mathrm{BCP}(s)}$
$4\mathrm{mm}1', 3\mathrm{m}1', 6\mathrm{mm}1'$	$\begin{pmatrix} 0 & 0 & 0 \\ 0 & 0 & 0 \\ xzx & 0 & 0 \end{pmatrix}$	$\begin{pmatrix} 0 & 0 & 0 \\ 0 & 0 & 0 \\ 0 & xzx & 0 \end{pmatrix}$	$\begin{pmatrix} -xzx & 0 & 0 \\ 0 & -xzx & 0 \\ 0 & 0 & zzz \end{pmatrix}$	$J^{\mathrm{BCP}(s)}$
$\bar{4}2\mathrm{m}1'$	$\begin{pmatrix} 0 & 0 & 0 \\ 0 & 0 & 0 \\ 0 & xzy & 0 \end{pmatrix}$	$\begin{pmatrix} 0 & 0 & 0 \\ 0 & 0 & 0 \\ xzy & 0 & 0 \end{pmatrix}$	$\begin{pmatrix} 0 & -xzy & 0 \\ -xzy & 0 & 0 \\ 0 & 0 & 0 \end{pmatrix}$	$K^{\mathrm{BCP}(s)}$

In this table, the candidate materials are not repeated, which are the same as the TABLE S13.

TABLE S18. Matrices expressions of second-order charge response tensor $\chi^{\mathrm{BCP(s)}}$ for Class III MPGs.

Class III MPGs	shortened form	Candidate materials
$2'$	$B^{\mathrm{BCP(s)}}$	$U_3Al_2Si_3$ [84]
m'	$C^{\mathrm{BCP(s)}}$	$U_3Al_2Si_3$ [25]
$22'2'$	$D^{\mathrm{BCP(s)}}$	(Cs,Rb)$NiCl_3$ [29, 30]
$2\mathrm{m}'\mathrm{m}', 2'\mathrm{mm}'$	$E^{\mathrm{BCP(s)}}$	$MnSO_4$ [32], $TbCrO_3$ [33],$MnSe_2$ [34]
$4', 6'$	$F^{\mathrm{BCP(s)}}$	$YMnO_3$ [37]
$\bar{4}'$	$H^{\mathrm{BCP(s)}}$	$CsCoF_4$ [38]
$4'22, 42'2', 32', 6'22', 62'2'$	$I^{\mathrm{BCP(s)}}$	$Er_2Ge_2O_7$ [42], Nd_5Si_4 [25], $NaMnFeF_6$ [43], $EuIn_2As_2$ [44], $DyMn_6Ge_6$ [45]
$4'\mathrm{mm}', 4\mathrm{m}'\mathrm{m}', 3\mathrm{m}', 6'\mathrm{mm}', 6\mathrm{m}'\mathrm{m}'$	$J^{\mathrm{BCP(s)}}$	Ce(Co,Ir)Ge_3 [48, 49], GaV_4S_8 [50], CrSe [85], (Yb,Ho,Sc)MnO_3 [18, 37, 51]
$\bar{4}'2\mathrm{m}', \bar{4}'2'\mathrm{m}, \bar{4}2'\mathrm{m}'$	$K^{\mathrm{BCP(s)}}$	Pb_2MnO_4 [53], Ce_4Sb_3 [54], $EuCr_2As_2$ [55], $MgCr_2O_4$ [56], li$FeCr_4O_8$ [72]

TABLE S19: Matrix representations of second-order spin response tensor $\chi^{\mathrm{D}(s)}$ for Class II MPGs.

Class II MPGs	χ_x^{D}	χ_y^{D}	χ_z^{D}	Shortened form	Candidate materials
$11'$	$\begin{pmatrix} xxx & xxy & xxz \\ xxy & xyy & xyz \\ xxz & xyz & xzz \end{pmatrix}$	$\begin{pmatrix} xxy & xyy & xyz \\ xyy & yyy & yyz \\ xyz & yyz & yzz \end{pmatrix}$	$\begin{pmatrix} xxz & xyz & zxz \\ xyz & yyz & yzz \\ xzz & yzz & zzz \end{pmatrix}$	$A^{\mathrm{D}(s)}$	$NiPS_3$ [24]
$21'$	$\begin{pmatrix} 0 & 0 & xxz \\ 0 & 0 & xyz \\ xxz & xyz & 0 \end{pmatrix}$	$\begin{pmatrix} 0 & 0 & xyz \\ 0 & 0 & yyz \\ xyz & yyz & 0 \end{pmatrix}$	$\begin{pmatrix} xxz & xyz & 0 \\ xyz & yyz & 0 \\ 0 & 0 & zzz \end{pmatrix}$	$B^{\mathrm{D}(s)}$	$HoNiO_3$ [11]
$\mathrm{m}1'$	$\begin{pmatrix} xxx & xxy & 0 \\ xxy & xyy & 0 \\ 0 & 0 & xzz \end{pmatrix}$	$\begin{pmatrix} xxy & xyy & 0 \\ xyy & yyy & 0 \\ 0 & 0 & yzz \end{pmatrix}$	$\begin{pmatrix} 0 & 0 & xzz \\ 0 & 0 & yzz \\ xzz & yzz & 0 \end{pmatrix}$	$C^{\mathrm{D}(s)}$	Na_2MnF_5 [26]
$231'$<br>$\bar{4}3\mathrm{m}1'$<br>$2221', \bar{4}2\mathrm{m}1'$	$\begin{pmatrix} 0 & 0 & 0 \\ 0 & 0 & xyz \\ 0 & xyz & 0 \end{pmatrix}$	$\begin{pmatrix} 0 & 0 & xyz \\ 0 & 0 & 0 \\ xyz & 0 & 0 \end{pmatrix}$	$\begin{pmatrix} 0 & xyz & 0 \\ xyz & 0 & 0 \\ 0 & 0 & 0 \end{pmatrix}$	$D^{\mathrm{D}(s)}$	–<br>$Gd_2Ti_2O_6$ [86]<br>$AgNiO_2$ [28], $GeCu_2O_4$ [52]
$2\mathrm{mm}1'$	$\begin{pmatrix} 0 & 0 & xxz \\ 0 & 0 & 0 \\ xxz & 0 & 0 \end{pmatrix}$	$\begin{pmatrix} 0 & 0 & 0 \\ 0 & 0 & yyz \\ 0 & yyz & 0 \end{pmatrix}$	$\begin{pmatrix} xxz & 0 & 0 \\ 0 & yyz & 0 \\ 0 & 0 & zzz \end{pmatrix}$	$E^{\mathrm{D}(s)}$	MnS_2 [31]
$41', 4\mathrm{mm}1', 61', 6\mathrm{mm}1'$	$\begin{pmatrix} 0 & 0 & xxz \\ 0 & 0 & 0 \\ xxz & 0 & 0 \end{pmatrix}$	$\begin{pmatrix} 0 & 0 & 0 \\ 0 & 0 & xxz \\ 0 & xxz & 0 \end{pmatrix}$	$\begin{pmatrix} xxz & 0 & 0 \\ 0 & xxz & 0 \\ 0 & 0 & zzz \end{pmatrix}$	$F^{\mathrm{D}(s)}$	$CeAuAl_3$ [35], CeAlGe [46]
$\bar{4}1'$	$\begin{pmatrix} 0 & 0 & xxz \\ 0 & 0 & xyz \\ xxz & xyz & 0 \end{pmatrix}$	$\begin{pmatrix} 0 & 0 & xyz \\ 0 & 0 & -xxz \\ xyz & -xxz & 0 \end{pmatrix}$	$\begin{pmatrix} xxz & xyz & 0 \\ xyz & -xxz & 0 \\ 0 & 0 & 0 \end{pmatrix}$	$H^{\mathrm{D}(s)}$	–
$321'$<br>$\bar{6}2\mathrm{m}1'$	$\begin{pmatrix} xxx & 0 & 0 \\ 0 & -xxx & 0 \\ 0 & 0 & 0 \end{pmatrix}$	$\begin{pmatrix} 0 & -xxx & 0 \\ -xxx & 0 & 0 \\ 0 & 0 & 0 \end{pmatrix}$	0	$I^{\mathrm{D}(s)}$	$SrFeO_3$ [40]<br>$Ba_6Co_6ClO_{15.5}$ [87]
$31'$	$\begin{pmatrix} xxx & -yyy & xxz \\ -yyy & -xxx & 0 \\ xxz & 0 & 0 \end{pmatrix}$	$\begin{pmatrix} -yyy & -xxx & 0 \\ -xxx & yyy & xxz \\ 0 & xxz & 0 \end{pmatrix}$	$\begin{pmatrix} xxz & 0 & 0 \\ 0 & xxz & 0 \\ 0 & 0 & zzz \end{pmatrix}$	$J^{\mathrm{D}(s)}$	Ni_3TeO_6 [36]
$3\mathrm{m}1'$	$\begin{pmatrix} 0 & -yyy & xxz \\ -yyy & 0 & 0 \\ xxz & 0 & 0 \end{pmatrix}$	$\begin{pmatrix} -yyy & 0 & 0 \\ 0 & yyy & xxz \\ 0 & xxz & 0 \end{pmatrix}$	$\begin{pmatrix} xxz & 0 & 0 \\ 0 & xxz & 0 \\ 0 & 0 & zzz \end{pmatrix}$	$K^{\mathrm{D}(s)}$	CuMnSb [47]
$\bar{6}1'$	$\begin{pmatrix} xxx & -yyy & 0 \\ -yyy & -xxx & 0 \\ 0 & 0 & 0 \end{pmatrix}$	$\begin{pmatrix} -yyy & -xxx & 0 \\ -xxx & yyy & 0 \\ 0 & 0 & 0 \end{pmatrix}$	0	$L^{\mathrm{D}(s)}$	TmPdIn [88]

TABLE S20: Matrix representations of second-order spin response tensor $\chi^{\mathrm{D}(s)}$ for Class III MPGs.

MPGs	$\chi_x^{\mathrm{D}(s)}$	$\chi_y^{\mathrm{D}(s)}$	$\chi_z^{\mathrm{D}(s)}$	Shortened form	Candidate materials
$2'$	$\begin{pmatrix} 0 & 0 & xxz \\ 0 & 0 & xyz \\ xxz & xyz & 0 \end{pmatrix}$	$\begin{pmatrix} 0 & 0 & xyz \\ 0 & 0 & yyz \\ xyz & yyz & 0 \end{pmatrix}$	$\begin{pmatrix} xxz & xyz & 0 \\ xyz & yyz & 0 \\ 0 & 0 & zzz \end{pmatrix}$	$B^{\mathrm{D}(s)}$	$U_3Al_2Si_3$ [84]
m'	$\begin{pmatrix} xxx & xxy & 0 \\ xxy & xyy & 0 \\ 0 & 0 & xzz \end{pmatrix}$	$\begin{pmatrix} xxy & xyy & 0 \\ xyy & yyy & 0 \\ 0 & 0 & yzz \end{pmatrix}$	$\begin{pmatrix} 0 & 0 & xzz \\ 0 & 0 & yzz \\ xzz & yzz & 0 \end{pmatrix}$	$C^{\mathrm{D}(s)}$	$U_3Al_2Si_3$ [25]
$22'2'$<br>$\bar{4}'3\mathrm{m}'$	$\begin{pmatrix} 0 & 0 & 0 \\ 0 & 0 & xyz \\ 0 & xyz & 0 \end{pmatrix}$	$\begin{pmatrix} 0 & 0 & xyz \\ 0 & 0 & 0 \\ xyz & 0 & 0 \end{pmatrix}$	$\begin{pmatrix} 0 & xyz & 0 \\ xyz & 0 & 0 \\ 0 & 0 & 0 \end{pmatrix}$	$D^{\mathrm{D}(s)}$	(Cs,Rb)$NiCl_3$ [29, 30]<br>−
$2\mathrm{m}'\mathrm{m}', 2'\mathrm{mm}'$	$\begin{pmatrix} 0 & 0 & xxz \\ 0 & 0 & 0 \\ xxz & 0 & 0 \end{pmatrix}$	$\begin{pmatrix} 0 & 0 & 0 \\ 0 & 0 & yyz \\ 0 & yyz & 0 \end{pmatrix}$	$\begin{pmatrix} xxz & 0 & 0 \\ 0 & yyz & 0 \\ 0 & 0 & zzz \end{pmatrix}$	$E^{\mathrm{D}(s)}$	$MnSO_4$ [32], $TbCrO_3$ [33]
$4', 4\mathrm{m}'\mathrm{m}'$<br>$4'\mathrm{mm}', 6'$<br>$6\mathrm{m}'\mathrm{m}', 6'\mathrm{mm}'$	$\begin{pmatrix} 0 & 0 & xxz \\ 0 & 0 & 0 \\ xxz & 0 & 0 \end{pmatrix}$	$\begin{pmatrix} 0 & 0 & 0 \\ 0 & 0 & xxz \\ 0 & xxz & 0 \end{pmatrix}$	$\begin{pmatrix} xxz & 0 & 0 \\ 0 & xxz & 0 \\ 0 & 0 & zzz \end{pmatrix}$	$F^{\mathrm{D}(s)}$	$YMnO_3$ [37], Ce(Co,Ir)Ge_3 [48, 49]<br>$YMnO_3$ [37]<br>(Yb,Ho,Sc)MnO_3 [18, 37, 51]
$\bar{4}'$	$\begin{pmatrix} 0 & 0 & xxz \\ 0 & 0 & xyz \\ xxz & xyz & 0 \end{pmatrix}$	$\begin{pmatrix} 0 & 0 & xyz \\ 0 & 0 & -xxz \\ xyz & -xxz & 0 \end{pmatrix}$	$\begin{pmatrix} xxz & xyz & 0 \\ xyz & -xxz & 0 \\ 0 & 0 & 0 \end{pmatrix}$	$H^{\mathrm{D}(s)}$	$CsCoF_4$ [38]
$\bar{6}'\mathrm{m}'2$	$\begin{pmatrix} xxx & 0 & 0 \\ 0 & -xxx & 0 \\ 0 & 0 & 0 \end{pmatrix}$	$\begin{pmatrix} 0 & -xxx & 0 \\ -xxx & 0 & 0 \\ 0 & 0 & 0 \end{pmatrix}$	0	$I^{\mathrm{D}(s)}$	$RbFeCl_3$ [68]
$\bar{6}'$	$\begin{pmatrix} xxx & -yyy & 0 \\ -yyy & -xxx & 0 \\ 0 & 0 & 0 \end{pmatrix}$	$\begin{pmatrix} -yyy & -xxx & 0 \\ -xxx & yyy & 0 \\ 0 & 0 & 0 \end{pmatrix}$	0	$L^{\mathrm{D}(s)}$	$Cu_{0.82}Mn_{1.18}As$ [63]
$\bar{4}2'\mathrm{m}', \bar{4}'2'\mathrm{m}$	$\begin{pmatrix} 0 & 0 & xxz \\ 0 & 0 & 0 \\ xxz & 0 & 0 \end{pmatrix}$	$\begin{pmatrix} 0 & 0 & 0 \\ 0 & 0 & -xxz \\ 0 & -xxz & 0 \end{pmatrix}$	$\begin{pmatrix} xxz & 0 & 0 \\ 0 & -xxz & 0 \\ 0 & 0 & 0 \end{pmatrix}$	$M^{\mathrm{D}(s)}$	$EuCr_2As_2$ [55], $MgCr_2O_4$ [56]
$32', \bar{6}\mathrm{m}'2'$<br>$\bar{6}'\mathrm{m}2'$	$\begin{pmatrix} 0 & -yyy & 0 \\ -yyy & 0 & 0 \\ 0 & 0 & 0 \end{pmatrix}$	$\begin{pmatrix} 0 & 0 & 0 \\ 0 & yyy & 0 \\ 0 & 0 & 0 \end{pmatrix}$	0	$N^{\mathrm{D}(s)}$	$NaMnFeF_6$ [43], $CsMnBr_3$ [82]<br>$CsFeCl_3$ [71]
$3\mathrm{m}'$	$\begin{pmatrix} xxx & 0 & xxz \\ 0 & -xxx & 0 \\ xxz & 0 & 0 \end{pmatrix}$	$\begin{pmatrix} 0 & -xxx & 0 \\ -xxx & 0 & xxz \\ 0 & xxz & 0 \end{pmatrix}$	$\begin{pmatrix} xxz & 0 & xzz \\ 0 & xxz & 0 \\ xzz & 0 & zzz \end{pmatrix}$	$O^{\mathrm{D}(s)}$	GaV_4S_8 [50], GaV_4S_8 [50]

C. Two-dimensional matrix representations for all response tensors

The second-order charge and spin response tensors can be expressed as $j_a^{(\sigma)} = \sum_{b,c} \chi_{abc}^{(\sigma)} E_b E_c$, where the χ_{abc} is a rank-three tensor and can be used a three-dimensional matrix to represent. Straightforwardly, we can also use a two-dimensional matrix to describe due to the commutative symmetry of the electric field components E_b and E_c. Hence, we blackefine a matrix to represent it [89],

$$\mathcal{E} = \begin{pmatrix} \mathcal{E}_1 \\ \mathcal{E}_2 \\ \mathcal{E}_3 \\ \mathcal{E}_4 \\ \mathcal{E}_5 \\ \mathcal{E}_6 \end{pmatrix} = \begin{pmatrix} E_x E_x \\ E_y E_y \\ E_z E_z \\ E_y E_z \\ E_x E_z \\ E_x E_y \end{pmatrix} \tag{S39}$$

The current density is

$$\begin{pmatrix} j_1^{(\sigma)} \\ j_2^{(\sigma)} \\ j_3^{(\sigma)} \end{pmatrix} = \begin{pmatrix} \chi_{11} & \chi_{12} & \chi_{13} & \chi_{14} & \chi_{15} & \chi_{16} \\ \chi_{21} & \chi_{22} & \chi_{23} & \chi_{24} & \chi_{25} & \chi_{26} \\ \chi_{31} & \chi_{32} & \chi_{33} & \chi_{34} & \chi_{35} & \chi_{36} \end{pmatrix} \begin{pmatrix} \mathcal{E}_1 \\ \mathcal{E}_2 \\ \mathcal{E}_3 \\ \mathcal{E}_4 \\ \mathcal{E}_5 \\ \mathcal{E}_6 \end{pmatrix} \tag{S40}$$

where $j_1^{(\sigma)}, j_2^{(\sigma)}, j_3^{(\sigma)} = j_x^{(\sigma)}, j_y^{(\sigma)}, j_z^{(\sigma)}$, the $\chi_{11}^{(\sigma)} = \chi_{xxx}^{(\sigma)}, \chi_{12}^{(\sigma)} = \chi_{xyy}^{(\sigma)}, \chi_{13}^{(\sigma)} = \chi_{xzz}^{(\sigma)}, \chi_{14}^{(\sigma)} = \chi_{xyz}^{(\sigma)} + \chi_{xzy}^{(\sigma)}, \chi_{15}^{(\sigma)} = \chi_{xzx}^{(\sigma)} + \chi_{xxz}^{(\sigma)}, \chi_{16}^{(\sigma)} = \chi_{xxy}^{(\sigma)} + \chi_{xyx}^{(\sigma)}$. Thus, the three term of the second conductivity can be written as

$$\chi^{BCD/BCP(\sigma)} = \begin{pmatrix} 0 & xyy & xzz & xyz + xzy & xzx & xyx \\ -xyx & 0 & yzz & yzy & -xyz + yzx & -xyy \\ -xzx & -yzy & 0 & -yzz & -xzz & -xzy - yzx \end{pmatrix} \tag{S41}$$

$$\chi^{D(\sigma)} = \begin{pmatrix} xxx & xyy & xzz & 2xyz & 2xxz & 2xxy \\ xxy & yyy & yzz & 2yyz & 2xyz & 2xyy \\ xxz & yyz & zzz & 2yzz & 2xzz & 2xyz \end{pmatrix} \tag{S42}$$

So, we give the two-dimensional matrix representations for the second-order charge and spin response tensor in the Table S21 and S23. This result is also completely consistent with the discussion about BCD term χ^{BCD}, which is χ^{in} in the Ref. [8] .

TABLE S21: Two-dimensional matrix representations for χ^{BCD} and $\chi^{\mathrm{BCD}(s)}$.

Shortened form	2-d matrix representations
$A^{\mathrm{BCD/BCD(s)}}$	$\begin{pmatrix} 0 & xyy & xzz & xyz+xzy & xzx & xyx \\ -xyx & 0 & yzz & yzy & -xyz+yzx & -xyy \\ -xzx & -yzy & 0 & -yzz & -xzz & -xzy-yzx \end{pmatrix}$
$B^{\mathrm{BCD/BCD(s)}}$	$\begin{pmatrix} 0 & 0 & 0 & xyz+xzy & xzx & 0 \\ 0 & 0 & 0 & yzy & -xyz+yzx & 0 \\ -xzx & -yzy & 0 & 0 & 0 & -xzy-yzx \end{pmatrix}$
$C^{\mathrm{BCD/BCD(s)}}$	$\begin{pmatrix} 0 & xyy & xzz & 0 & 0 & xyx \\ -xyx & 0 & yzz & 0 & 0 & -xyy \\ 0 & 0 & 0 & -yzz & -xzz & 0 \end{pmatrix}$
$D^{\mathrm{BCD/BCD(s)}}$	$\begin{pmatrix} 0 & 0 & 0 & xyz+xzy & 0 & 0 \\ 0 & 0 & 0 & 0 & -xyz+yzx & 0 \\ 0 & 0 & 0 & 0 & 0 & -xzy-yzx \end{pmatrix}$
$E^{\mathrm{BCD/BCD(s)}}$	$\begin{pmatrix} 0 & 0 & 0 & 0 & xzx & 0 \\ 0 & 0 & 0 & yzy & 0 & 0 \\ -xzx & -yzy & 0 & 0 & 0 & 0 \end{pmatrix}$
$F^{\mathrm{BCD/BCD(s)}}$	$\begin{pmatrix} 0 & 0 & 0 & xyz+xzy & xzx & 0 \\ 0 & 0 & 0 & xzx & -xyz-xzy & 0 \\ -xzx & -xzx & 0 & 0 & 0 & 0 \end{pmatrix}$
$H^{\mathrm{BCD/BCD(s)}}$	$\begin{pmatrix} 0 & 0 & 0 & xzy & xzx & 0 \\ 0 & 0 & 0 & -xzx & xzy & 0 \\ -xzx & xzx & 0 & 0 & 0 & -2xzy \end{pmatrix}$
$I^{\mathrm{BCD/BCD(s)}}$	$\begin{pmatrix} 0 & 0 & 0 & xyz+xzy & 0 & 0 \\ 0 & 0 & 0 & 0 & -xyz-xzy & 0 \\ 0 & 0 & 0 & 0 & 0 & 0 \end{pmatrix}$
J^{BCD}	$\begin{pmatrix} 0 & 0 & 0 & 0 & xzx & 0 \\ 0 & 0 & 0 & xzx & 0 & 0 \\ -xzx & -xzx & 0 & 0 & 0 & 0 \end{pmatrix}$
K^{BCD}	$\begin{pmatrix} 0 & 0 & 0 & xzy & 0 & 0 \\ 0 & 0 & 0 & 0 & xzy & 0 \\ 0 & 0 & 0 & 0 & 0 & -2xzy \end{pmatrix}$
$L^{\mathrm{BCD}(s)}$	$\begin{pmatrix} 0 & xyy & xzz & 0 & 0 & 0 \\ 0 & 0 & 0 & 0 & 0 & -xyy \\ 0 & 0 & 0 & 0 & -xzz & 0 \end{pmatrix}$
$M^{\mathrm{BCD}(s)}$	$\begin{pmatrix} 0 & 0 & 0 & 0 & xzx & 0 \\ 0 & 0 & 0 & xzx & 0 & 0 \\ -xzx & -xzx & 0 & 0 & 0 & 0 \end{pmatrix}$
$N^{\mathrm{BCD}(s)}$	$\begin{pmatrix} 0 & 0 & 0 & xzy & 0 & 0 \\ 0 & 0 & 0 & 0 & -xzy & 0 \\ 0 & 0 & 0 & 0 & 0 & 0 \end{pmatrix}$
$O^{\mathrm{BCD}(s)}$	$\begin{pmatrix} 0 & 0 & 0 & 0 & xzx & 0 \\ 0 & 0 & 0 & -xzx & 0 & 0 \\ -xzx & xzx & 0 & 0 & 0 & 0 \end{pmatrix}$

TABLE S22: Two-dimensional matrix representations for χ^{BCP} and $\chi^{\mathrm{BCP}(s)}$.

Shortened form	2-d matrix representations
$A^{\mathrm{BCP/BCP}(s)}$	$\begin{pmatrix} 0 & xyy & xzz & xyz+xzy & xzx & xyx \\ -xyx & 0 & yzz & yzy & -xyz+yzx & -xyy \\ -xzx & -yzy & 0 & -yzz & -xzz & -xzy-yzx \end{pmatrix}$
$B^{\mathrm{BCP/BCP}(s)}$	$\begin{pmatrix} 0 & 0 & 0 & xyz+xzy & xzx & 0 \\ 0 & 0 & 0 & yzy & -xyz+yzx & 0 \\ -xzx & -yzy & 0 & 0 & 0 & -xzy-yzx \end{pmatrix}$
$C^{\mathrm{BCP/BCP(s)}}$	$\begin{pmatrix} 0 & xyy & xzz & 0 & 0 & xyx \\ -xyx & 0 & yzz & 0 & 0 & -xyy \\ 0 & 0 & 0 & -yzz & -xzz & 0 \end{pmatrix}$
$D^{\mathrm{BCP/BCP(s)}}$	$\begin{pmatrix} 0 & 0 & 0 & xyz+xzy & 0 & 0 \\ 0 & 0 & 0 & 0 & -xyz+yzx & 0 \\ 0 & 0 & 0 & 0 & 0 & -xzy-yzx \end{pmatrix}$
$E^{\mathrm{BCP/BCP(s)}}$	$\begin{pmatrix} 0 & 0 & 0 & 0 & xzx & 0 \\ 0 & 0 & 0 & yzy & 0 & 0 \\ -xzx & -yzy & 0 & 0 & 0 & 0 \end{pmatrix}$
$F^{\mathrm{BCP/BCP(s)}}$	$\begin{pmatrix} 0 & 0 & 0 & xyz+xzy & xzx & 0 \\ 0 & 0 & 0 & xzx & -xyz-xzy & 0 \\ -xzx & -xzx & 0 & 0 & 0 & 0 \end{pmatrix}$
$H^{\mathrm{BCP/BCP(s)}}$	$\begin{pmatrix} 0 & 0 & 0 & xzy & xzx & 0 \\ 0 & 0 & 0 & -xzx & xzy & 0 \\ -xzx & xzx & 0 & 0 & 0 & -2xzy \end{pmatrix}$
$I^{\mathrm{BCP/BCP(s)}}$	$\begin{pmatrix} 0 & 0 & 0 & xyz+xzy & 0 & 0 \\ 0 & 0 & 0 & 0 & -xyz-xzy & 0 \\ 0 & 0 & 0 & 0 & 0 & 0 \end{pmatrix}$
$J^{\mathrm{BCP/BCP(s)}}$	$\begin{pmatrix} 0 & 0 & 0 & 0 & xzx & 0 \\ 0 & 0 & 0 & xzx & 0 & 0 \\ -xzx & -xzx & 0 & 0 & 0 & 0 \end{pmatrix}$
$K^{\mathrm{BCP/BCP(s)}}$	$\begin{pmatrix} 0 & 0 & 0 & xzy & 0 & 0 \\ 0 & 0 & 0 & 0 & xzy & 0 \\ 0 & 0 & 0 & 0 & 0 & -2xzy \end{pmatrix}$

TABLE S23: Two-dimensional matrix representations for χ^{D} and $\chi^{\mathrm{D}(s)}$.

Shortened form	2-d matrix representations
$A^{\mathrm{D/D}(s)}$	$\begin{pmatrix} xxx & xyy & xzz & 2xyz & 2xxz & 2xxy \\ xxy & yyy & yzz & 2yyz & 2xyz & 2xyy \\ xxz & yyz & zzz & 2yzz & 2xzz & 2xyz \end{pmatrix}$
$B^{\mathrm{D/D}(s)}$	$\begin{pmatrix} 0 & 0 & 0 & 2xyz & 2xxz & 0 \\ 0 & 0 & 0 & 2yyz & 2xyz & 0 \\ xxz & yyz & zzz & 0 & 0 & 2xyz \end{pmatrix}$
$C^{\mathrm{D/D}(s)}$	$\begin{pmatrix} xxx & xyy & xzz & 0 & 0 & 2xxy \\ xxy & yyy & yzz & 0 & 0 & 2xyy \\ 0 & 0 & 0 & 2yzz & 2xzz & 0 \end{pmatrix}$
$D^{\mathrm{D/D}(s)}$	$\begin{pmatrix} 0 & 0 & 0 & 2xyz & 0 & 0 \\ 0 & 0 & 0 & 0 & 2xyz & 0 \\ 0 & 0 & 0 & 0 & 0 & 2xyz \end{pmatrix}$
$E^{\mathrm{D/D}(s)}$	$\begin{pmatrix} 0 & 0 & 0 & 0 & 2xxz & 0 \\ 0 & 0 & 0 & 2yyz & 0 & 0 \\ xxz & yyz & zzz & 0 & 0 & 0 \end{pmatrix}$
$F^{\mathrm{D/D}(s)}$	$\begin{pmatrix} 0 & 0 & 0 & 0 & 2xxz & 0 \\ 0 & 0 & 0 & 2xxz & 0 & 0 \\ xxz & xxz & zzz & 0 & 0 & 0 \end{pmatrix}$
$H^{\mathrm{D/D}(s)}$	$\begin{pmatrix} 0 & 0 & 0 & 2xyz & 2xxz & 0 \\ 0 & 0 & 0 & -2xxz & 2xyz & 0 \\ xxz & -xxz & 0 & 0 & 0 & 2xyz \end{pmatrix}$
$I^{\mathrm{D/D}(s)}$	$\begin{pmatrix} xxx & -xxx & 0 & 0 & 0 & 0 \\ 0 & 0 & 0 & 0 & 0 & -2xxx \\ 0 & 0 & 0 & 0 & 0 & 0 \end{pmatrix}$
$J^{\mathrm{D/D}(s)}$	$\begin{pmatrix} xxx & -xxx & 0 & 0 & 2xxz & -2yyy \\ -yyy & yyy & 0 & 2xxz & 0 & -2xxx \\ xxz & xxz & zzz & 0 & 0 & 0 \end{pmatrix}$
$K^{\mathrm{D/D}(s)}$	$\begin{pmatrix} 0 & 0 & 0 & 0 & 2xxz & -2yyy \\ -yyy & yyy & 0 & 2xxz & 0 & 0 \\ xxz & xxz & zzz & 0 & 0 & 0 \end{pmatrix}$
$L^{\mathrm{D/D}(s)}$	$\begin{pmatrix} xxx & -xxx & 0 & 0 & 0 & -2yyy \\ -yyy & yyy & 0 & 0 & 0 & -2xxx \\ 0 & 0 & 0 & 0 & 0 & 0 \end{pmatrix}$
$M^{\mathrm{D}(s)}$	$\begin{pmatrix} 0 & 0 & 0 & 0 & 2xxz & 0 \\ 0 & 0 & 0 & -2xxz & 0 & 0 \\ xxz & -xxz & 0 & 0 & 0 & 0 \end{pmatrix}$
$N^{\mathrm{D}(s)}$	$\begin{pmatrix} 0 & 0 & 0 & 0 & 0 & -2yyy \\ -yyy & yyy & 0 & 0 & 0 & 0 \\ 0 & 0 & 0 & 0 & 0 & 0z \end{pmatrix}$
$O^{\mathrm{D}(s)}$	$\begin{pmatrix} xxx & -xxx & 0 & 0 & 2xxz & 0 \\ 0 & 0 & 0 & 2xxz & 0 & -2xxx \\ xxz & xxz & 0 & 0 & 0 & 0 \end{pmatrix}$

* zgzhu@ucas.ac.cn
† gsu@ucas.ac.cn

[1] Inti Sodemann and Liang Fu, "Quantum nonlinear hall effect induced by berry curvature dipole in time-reversal invariant materials," Phys. Rev. Lett. **115**, 216806 (2015).
[2] Yang Gao, Shengyuan A. Yang, and Qian Niu, "Field induced positional shift of bloch electrons and its dynamical implications," Phys. Rev. Lett. **112**, 166601 (2014).
[3] Chong Wang, Yang Gao, and Di Xiao, "Intrinsic nonlinear hall effect in antiferromagnetic tetragonal CuMnAs," Phys. Rev. Lett. **127**, 277201 (2021).
[4] Huiying Liu, Jianzhou Zhao, Yue-Xin Huang, Weikang Wu, Xian-Lei Sheng, Cong Xiao, and Shengyuan A. Yang, "Intrinsic second-order anomalous hall effect and its application in compensated antiferromagnets," Phys. Rev. Lett. **127**, 277202 (2021).
[5] Yang Gao, "Semiclassical dynamics and nonlinear charge current," Front. Phys. **14** (2019), 10.1007/s11467-019-0887-2.
[6] J. Železný, Z. Fang, K. Olejník, J. Patchett, F. Gerhard, C. Gould, L. W. Molenkamp, C. Gomez-Olivella, J. Zemen, T. Tichý, T. Jungwirth, and C. Ciccarelli, "Unidirectional magnetoresistance and spin-orbit torque in NiMnSb," Phys. Rev. B **104**, 054429 (2021).
[7] C. L. Kane and E. J. Mele, "Quantum spin hall effect in graphene," Phys. Rev. Lett. **95**, 226801 (2005).
[8] Z. Z. Du, C. M. Wang, Hai-Peng Sun, Hai-Zhou Lu, and X. C. Xie, "Quantum theory of the nonlinear hall effect," Nat Commun **12**, 5038 (2021).
[9] Satoru Hayami, Megumi Yatsushiro, and Hiroaki Kusunose, "Nonlinear spin hall effect in PT-symmetric collinear magnets," Phys. Rev. B **106**, 024405 (2022).
[10] Cheng-Ping Zhang, Jiewen Xiao, Benjamin T. Zhou, and Jin-Xin, "Giant nonlinear hall effect in strained twisted bilayer graphene," arXiv:2010.08333 (2022).
[11] M. T. Fernández-Díaz, J. A. Alonso, M. J. Martínez-Lope, M. T. Casais, and J. L. García-Muñoz, "Magnetic structure of the $HoNiO_3$ perovskite," Phys. Rev. B **64**, 144417 (2001).
[12] Z. Z. Du, C. M. Wang, Hai-Zhou Lu, and X. C. Xie, "Band signatures for strong nonlinear hall effect in bilayer WTe_2," Phys. Rev. Lett. **121**, 266601 (2018).
[13] Raffaele Battilomo, Niccoló Scopigno, and Carmine Ortix, "Berry curvature dipole in strained graphene: A fermi surface warping effect," Phys. Rev. Lett. **123**, 196403 (2019).
[14] G. Rousse, J. Rodriguez-Carvajal, S. Patoux, and C. Masquelier, "Magnetic structures of the triphylite $LiFePO_4$ and of its delithiated form $FePO_4$," Chem. Mater. **15**, 4082–4090 (2003).
[15] J. A. Gonzalo, D. E. Cox, and G. Shirane, "The magnetic structure of $FeSb_2O_4$," Phys. Rev. **147**, 415–418 (1966).
[16] Stanislav Podchezertsev, Nicolas Barrier, Alain Pautrat, Emmanuelle Suard, María Retuerto, Jose Antonio Alonso, María Teresa Fernández-Díaz, and Juan Rodríguez-Carvajal, "Influence of polymorphism on the magnetic properties of co_5TeO_8 spinel," Inorg. Chem. **60**, 13990–14001 (2021).
[17] Jan-Willem G. Bos, Claire V. Colin, and Thomas T. M. Palstra, "Magnetoelectric coupling in the cubic ferrimagnet Cu_2OSeO_3," Phys. Rev. B **78**, 094416 (2008).
[18] A. Muñoz, J. A. Alonso, M. J. Martínez-Lope, M. T. Casáis, J. L. Martínez, and M. T. Fernández-Díaz, "Magnetic structure of hexagonal $RMnO_3$(R=Y,Sc): Thermal evolution from neutron powder diffraction data." Phys. Rev. B **62**, 9498–9510 (2000).
[19] E. Morosan, J. A. Fleitman, Q. Huang, J. W. Lynn, Y. Chen, X. Ke, M. L. Dahlberg, P. Schiffer, C. R. Craley, and R. J. Cava, "Structure and magnetic properties of the $Ho_2Ge_2O_7$ pyrogermanate," Phys. Rev. B **77**, 224423 (2008).
[20] F. Li, V. Pomjakushin, T. Mazet, R. Sibille, B. Malaman, R. Yadav, L. Keller, M. Medarde, K. Conder, and E. Pomjakushina, "Revisiting the magnetic structure and charge ordering in $La_{1/3}Sr_{2/3}FeO_3$ by neutron powder diffraction and mssbauer spectroscopy," Phys. Rev. B **97**, 174417 (2018).
[21] X. F. Hao, A. Stroppa, S. Picozzi, A. Filippetti, and C. Franchini, "Exceptionally large room-temperature ferroelectric poloarizability in the $PbNiO_3$ multiferroic nickelate: First-principles study," Phys. Rev. B **86**, 014116 (2012).
[22] A. Muñoz, J. A. Alonso, M. J. Martínez-Lope, M. T. Casáis, J. L. Martínez, and M. T. Fernández-Díaz, "Evolution of the magnetic structure of hexagonal $HoMnO_3$ from neutron powder diffraction data," Chem. Mater. **13**, 1497–1505 (2001).
[23] Cheng Chen, Huaiqiang Wang, Dinghui Wang, and Haijun Zhang, "Strain-engineeblack nonlinear hall effect in HgTe," SPIN **09**, 1940017 (2019).
[24] A. R. Wildes, V. Simonet, E. Ressouche, G. J. McIntyre, M. Avdeev, E. Suard, S. A. J. Kimber, D. Lançon, G. Pepe, B. Moubaraki, and T. J. Hicks, "Magnetic structure of the quasi-two-dimensional antiferromagnet $NiPS_3$," Phys. Rev. B **92**, 224408 (2015).
[25] J M Cadogan, D H Ryan, Z Altounian, H B Wang, and I P Swainson, "The magnetic structures of Nd_5Si_4 and Nd_5Ge_4," J. Phys.: Condens. Matter **14**, 7191–7200 (2002).
[26] P. Núñez, T. Roisnel, and A. Tressaud, "Magnetic structure of the chain mn(III) fluoride Na_2MnF_5," Solid State Commun. **92**, 601–605 (1994).
[27] Angel M. Arévalo-López and J. Paul Attfield, "Weak ferromagnetism and domain effects in multiferroic $LiNbO_3$-type $MnTiO_3$." Phys. Rev. B **88**, 104416 (2013).
[28] E. Wawrzyńska, R. Coldea, E. M. Wheeler, T. Srgel, M. Jansen, R. M. Ibberson, P. G. Radaelli, and M. M. Koza, "Charge disproportionation and collinear magnetic order in the frustrated triangular antiferromagnet $AgNiO_2$," Phys. Rev. B **77**,

094439 (2008).
[29] W. B. Yelon and D. E. Cox, "Magnetic transitions in $CsNi_3$." Phys. Rev. B **7**, 2024–2027 (1973).
[30] V.J. Minkiewicz, D.E. Cox, and G. Shirane, "The magnetic structures of $RbNiCl_3$ and $CsNiCl_3$." Solid State Commun. **8**, 1001–1005 (1970).
[31] L. M. Corliss, N. Elliott, and J. M. Hastings, "Antiferromagnetic structures of MnS_2, $MnSe_2$ and $MnTe_2$," J. Appl. Phys. **29**, 391–392 (1958).
[32] G. Will, B. C. Frazer, G. Shirane, D. E. Cox, and P. J. Brown, "Magnetic structure of MnS_4." Phys. Rev. **140**, A2139–A2142 (1965).
[33] E.F. Bertaut, J. Chappert, J. Mareschal, J.P. Rebouillat, and J. Sivardière, "Structures magnetiques de $TbFeO_3$," Solid State Commun. **5**, 293–298 (1967).
[34] T. Chattopadhyay, J. Rossat-Mignod, and H. Fjellvåg, "Magnetic ordering in $MnSe_2$," Solid State Commun. **63**, 65–67 (1987).
[35] D. T. Adroja, C. de la Fuente, A. Fraile, A. D. Hillier, A. Daoud-Aladine, W. Kockelmann, J. W. Taylor, M. M. Koza, E. Burzurí, F. Luis, J. I. Arnaudas, and A. del Moral, "Muon spin rotation and neutron scattering study of the noncentrosymmetric tetragonal compound $CeAuAl_3$," Phys. Rev. B **91**, 134425 (2015).
[36] I Živković, K Prša, O Zaharko, and H Berger, "Ni_3TeO_6—a collinear antiferromagnet with ferromagnetic honeycomb planes," J. Phys.: Condens. Matter **22**, 056002 (2010).
[37] P J Brown and T Chatterji, "Neutron diffraction and polarimetric study of the magnetic and crystal structures of $HoMnO_3$ and $YMnO_3$." J. Phys.: Condens. Matter **18**, 10085–10096 (2006).
[38] P. Lacorre, J. Pannetier, T. Fleischer, R. Hoppe, and G. Ferey, "Ordeblack magnetic frustration: XVI. magnetic structure of $cscof_4$ at 1.5 k," J. Solid State Chem. **93**, 37–45 (1991).
[39] Hirohiko Sato, Koji Wakiya, Toshiaki Enoki, Takashi Kiyama, Yusuke Wakabayashi, Hironori Nakao, and Youichi Murakami, "Magnetic structure of β-MnO_2: X-ray magnetic scattering study," J. Phys. Soc. Jpn. **70**, 37–40 (2001).
[40] M. Reehuis, C. Ulrich, A. Maljuk, Ch. Niedermayer, B. Ouladdiaf, A. Hoser, T. Hofmann, and B. Keimer, "Neutron diffraction study of spin and charge ordering in $SrFeO_{3-\delta}$," Phys. Rev. B **85**, 184109 (2012).
[41] P. Schobinger-Papamantellos, J. Rodríguez-Carvajal, and K.H.J. Buschow, "Magnetic ordering of $ScMn_6Ge_6$ by neutron diffraction," J. Magn. Magn. Mater. **369**, 243–248 (2014).
[42] K. M. Taddei, L. Sanjeewa, J. W. Kolis, A. S. Sefat, C. de la Cruz, and D. M. Pajerowski, "Local-ising-type magnetic order and metamagnetism in the rare-earth pyrogermanate $Er_2Ge_2O_7$," Phys. Rev. Materials **3**, 014405 (2019).
[43] G. Courbion and M. Leblanc, "The magnetic structure of $NaMnFeF_6$," J. Magn. Magn. Mater. **74**, 158–164 (1988).
[44] S. X. M. Riberolles, T. V. Trevisan, B. Kuthanazhi, T. W. Heitmann, F. Ye, D. C. Johnston, S. L. Bud'ko, D. H. Ryan, P. C. Canfield, A. Kreyssig, A. Vishwanath, R. J. McQueeney, L. L. Wang, P. P. Orth, and B. G. Ueland, "Magnetic crystalline-symmetry-protected axion electrodynamics and field-tunable unpinned dirac cones in $EuIn_2As_2$," Nat Commun **12** (2021), 10.1038/s41467-021-21154-y.
[45] J. Rodríguez-Carvajal and F. Bourée, "Symmetry and magnetic structures," EPJ Web of Conferences **22**, 00010 (2012).
[46] Pascal Puphal, Vladimir Pomjakushin, Naoya Kanazawa, Victor Ukleev, Dariusz J. Gawryluk, Junzhang Ma, Muntaser Naamneh, Nicholas C. Plumb, Lukas Keller, Robert Cubitt, Ekaterina Pomjakushina, and Jonathan S. White, "Topological magnetic phase in the candidate weyl semimetal CeAlGe," Phys. Rev. Lett. **124**, 017202 (2020).
[47] A. Regnat, A. Bauer, A. Senyshyn, M. Meven, K. Hradil, P. Jorba, K. Nemkovski, B. Pedersen, R. Georgii, S. Gottlieb-Schnmeyer, and C. Pfleiderer, "Canted antiferromagnetism in phase-pure CuMnSb," Phys. Rev. Materials **2**, 054413 (2018).
[48] M. Smidman, D. T. Adroja, A. D. Hillier, L. C. Chapon, J. W. Taylor, V. K. Anand, R. P. Singh, M. R. Lees, E. A. Goremychkin, M. M. Koza, V. V. Krishnamurthy, D. M. Paul, and G. Balakrishnan, "Neutron scattering and muon spin relaxation measurements of the noncentrosymmetric antiferromagnet $CeCoGe_3$," Phys. Rev. B **88**, 134416 (2013).
[49] V. K. Anand, A. D. Hillier, D. T. Adroja, D. D. Khalyavin, P. Manuel, G. Andre, S. Rols, and M. M. Koza, "Understanding the magnetism in noncentrosymmetric $CeIrGe_3$: Muon spin relaxation and neutron scattering studies." Phys. Rev. B **97**, 184422 (2018).
[50] S J R Holt, C Ritter, M R Lees, and G Balakrishnan, "Investigation of the magnetic ground state of gav_4s_8." J. Phys.: Condens. Matter **33**, 255802 (2021).
[51] X. Fabrèges, I. Mirebeau, P. Bonville, S. Petit, G. Lebras-Jasmin, A. Forget, G. André, and S. Pailhès, "Magnetic order in $YbMnO_3$ studied by neutron diffraction and mssbauer spectroscopy." Phys. Rev. B **78**, 214422 (2008).
[52] T. Zou, Y.-Q. Cai, C. R. dela Cruz, V. O. Garlea, S. D. Mahanti, J.-G. Cheng, and X. Ke, "Up-up-down-down magnetic chain structure of the spin- 1/2 tetragonally distorted spinel $GeCu_2O_4$," Phys. Rev. B **94**, 214406 (2016).
[53] Simon A. J. Kimber and J. Paul Attfield, "Magnetic order in acentric Pb_2MnO_4," J. Mater. Chem. **17**, 4885 (2007).
[54] R. Nirmala, A.V. Morozkin, O. Isnard, and A.K. Nigam, "Understanding the magnetic ground state of rare-earth intermetallic compound Ce_4Sb_3: Magnetization and neutron diffraction studies," J. Magn. Magn. Mater. **321**, 188–191 (2009).
[55] S. Nandi, Y. Xiao, N. Qureshi, U. B. Paramanik, W. T. Jin, Y. Su, B. Ouladdiaf, Z. Hossain, and Th. Brckel, "Magnetic structures of the eu and cr moments in $EuCr_2As_2$: Neutron diffraction study." Phys. Rev. B **94**, 094411 (2016).
[56] H. Shaked, J. M. Hastings, and L. M. Corliss, "Magnetic structure of magnesium chromite," Phys. Rev. B **1**, 3116–3124 (1970).
[57] E.F. Bertaut, J. Cohen, B. Lambert-Andron, and P. Mollard, "Étude de Cr_2S_3 rhomboédrique par diffraction neutronique et mesures magnétiques," J. Phys. **29**, 813–824 (1968).

[58] P. Schobinger-Papamantellos, G. André, J. Rodríguez-Carvajal, C.H. de Groot, and K.H.J. Buschow, "The magnetic ordering of the novel compound $ErGe_3$," J. Alloys **232**, 165–168 (1996).
[59] M. Fiebig, D. Frhlich, and H. J. Thiele, "Determination of spin direction in the spin-flop phase of Cr_2O_3," Phys. Rev. B **54**, R12681–R12684 (1996).
[60] D Sheptyakov, N Z Ali, and M Jansen, "A neutron diffraction study of structural and magnetic transformations in $AFeO_2$(a = K, Rb and Cs)," J. Phys.: Condens. Matter **22**, 426001 (2010).
[61] Hiroki Yamauchi, Naoto Metoki, Ryuta Watanuki, Kazuya Suzuki, Hiroshi Fukazawa, Songxue Chi, and Jaime A. Fernandez-Baca, "Magnetic structure and quadrupolar order parameter driven by geometrical frustration effect in NdB_4," J. Phys. Soc. Jpn. **86**, 044705 (2017).
[62] Gen Shirane, S. J. Pickart, and Yoshikazu Ishikawa, "Neutron diffraction study of antiferromagnetic $MnTiO_3$ and $NiTiO_3$," J. Phys. Soc. Jpn. **14**, 1352–1360 (1959).
[63] Manohar H. Karigerasi, Kisung Kang, Arun Ramanathan, Danielle L. Gray, Matthias D. Frontzek, Huibo Cao, André Schleife, and Daniel P. Shoemaker, "In-plane hexagonal antiferromagnet in the Cu-Mn-As system $Cu_{0.82}Mn_{1.18}As$," Phys. Rev. Materials **3**, 111402 (2019).
[64] P J Brown, J Crangle, K-U Neumann, J G Smith, and K R A Ziebeck, "The structure and magnetic moment distribution in the antiferromagnetic phase of $Cu_{14}Au_{51}$," J. Phys.: Condens. Matter **9**, 4729–4742 (1997).
[65] Sean Injac, Alexander K. L. Yuen, Maxim Avdeev, Fabio Orlandi, and Brendan J. Kennedy, "Structural and magnetic studies of $KOsO_4$,a $5d^1$ quantum magnet oxide," Phys. Chem. Chem. Phys. **21**, 7261–7264 (2019).
[66] J Konstantinovic, G Stanisic, M Ain, and G Parette, "On the magnetic structure of Bi_2CuO_4," J. Phys.: Condens. Matter **3**, 381–384 (1991).
[67] P J Brown, J B Forsyth, E Lelièvre-Berna, and F Tasset, "Determination of the magnetization distribution in Cr_2O_3 using spherical neutron polarimetry," J. Phys.: Condens. Matter **14**, 1957–1966 (2002).
[68] G. R. Davidson, M. Eibschutz, D. E. Cox, V. J. Minkiewicz, C. D. Graham, and J. J. Rhyne, "MAGNETIC ORDERING IN a LINEAR CHAIN COMPOUND $RbFeCl_3$," in *AIP Conference Proceedings* (AIP, 1972).
[69] Q. Cui, Q. Huang, Jose A. Alonso, Denis Sheptyakov, C. R. De la Cruz, M. T. Fernández-Díaz, N. N. Wang, Y. Q. Cai, D. Li, X. L. Dong, H. D. Zhou, and J.-G. Cheng, "Complex antiferromagnetic order in the garnet $Co_3Al_2Si_3O_{12}$," Phys. Rev. B **101**, 144424 (2020).
[70] JoAnna Milam-Guerrero, Michelle Zheng, Nicole R. Spence, Mario Falsaperna, Stuart Calder, Saul Lapidus, Paul J. Saines, and Brent C. Melot, "Influence of the cubic sublattice on magnetic coupling between the tetrahedral sites of garnet," Inorg. Chem. **60**, 8500–8506 (2021).
[71] Shohei Hayashida, Oksana Zaharko, Nobuyuki Kurita, Hidekazu Tanaka, Masato Hagihala, Minoru Soda, Shinichi Itoh, Yoshiya Uwatoko, and Takatsugu Masuda, "Pressure-induced quantum phase transition in the quantum antiferromagnet $CsFeCl_3$," Phys. Rev. B **97**, 140405 (2018).
[72] Rana Saha, R. Dhanya, Christophe Bellin, Keevin Béneut, Arpan Bhattacharyya, A. Shukla, Chandrabhas Narayana, E. Suard, J. Rodríguez-Carvajal, and A. Sundaresan, "Magnetostructural coupling and magnetodielectric effects in the $LiFeCr_4O_8$." Phys. Rev. B **96**, 214439 (2017).
[73] B. Roy, Abhishek Pandey, Q. Zhang, T. W. Heitmann, D. Vaknin, D. C. Johnston, and Y. Furukawa, "Experimental evidence of a collinear antiferromagnetic ordering in the frustrated $CoAl_2O_4$," Phys. Rev. B **88**, 174415 (2013).
[74] Elena Solana-Madruga, Clemens Ritter, Cintli Aguilar-Maldonado, Olivier Mentré, J. Paul Attfield, and Ángel M. Arévalo-López, "$MnMn_3Nb_2O_9$: high-pressure triple perovskite with 1:2 B-site order and modulated spins," Chem. Commun. **57**, 8441–8444 (2021).
[75] A.V. Morozkin, O. Isnard, R. Nirmala, and S.K. Malik, "Magnetic structure of the rare earth intermetallic compound Ce_4Ge_3," J. Alloys **486**, 497–500 (2009).
[76] T. Eriksson, R. Lizárraga, S. Felton, L. Bergqvist, Y. Andersson, P. Nordblad, and O. Eriksson, "Crystal and magnetic structure of Mn_3IrGe," Phys. Rev. B **69**, 054422 (2004).
[77] T. Eriksson, L. Bergqvist, P. Nordblad, O. Eriksson, and Y. Andersson, "Structural and magnetic characterization of Mn_3IrGe and $Mn_3IrSi_{1-x}Ge_x$: experiments and theory," J. Solid State Chem. **177**, 4058–4066 (2004).
[78] Yuki M. Itahashi, Toshiya Ideue, Shintaro Hoshino, Chihiro Goto, H. Namiki, T. Sasagawa, and Yoshihiro Iwasa, "Giant intrinsic rectification and nonlinear hall effect under time-reversal symmetry in a trigonal superconductor," arXiv:2202.11876 (2022).
[79] Edouard Lesne, Yildiz G. Salam, Raffaele Battilomo, Thierry C. van Thiel, Ulderico Filippozzi, Mario Cuoco, Gary A. Steele, Carmine Ortix, and Andrea D. Caviglia, "Designing berry curvature dipoles and the quantum nonlinear hall effect at oxide interfaces," arXiv:2201.12161 (2022), arXiv:2201.12161.
[80] A. Samartzis, S. Chillal, A. T. M. N. Islam, K. Siemensmeyer, K. Prokes, D. J. Voneshen, A. Senyshyn, D. Khalyavin, and B. Lake, "Structural and magnetic properties of the quantum magnet $BaCuTe_2O_6$," Phys. Rev. B **103**, 094417 (2021).
[81] V.M.T.S. Barthem, C.V. Colin, H. Mayaffre, M.-H. Julien, and D. Givord, "Revealing the properties of Mn_2Au for antiferromagnetic spintronics," Nat Commun **4** (2013), 10.1038/ncomms3892.
[82] M. Eibschutz, R. C. Sherwood, F. S. L. Hsu, C. E. Cox, Hugh C. Wolfe, C. D. Graham, and J. J. Rhyne, "Magnetic ordering of the linear chain antiferromagnet $CsMnBr_3$," in *AIP Conference Proceedings* (AIP, 1973).
[83] A. Dommann, H.R. Ott, F. Hulliger, and P. Fischer, "The crystal structure and the magnetic order of $U_{14}Au_{51}$," Journal of the Less Common Metals **160**, 171–180 (1990).
[84] A G Gukasov, P Rogl, P J Brown, M Mihalik, and A Menovsky, "Site susceptibility tensors and magnetic structure of $U_3Al_2Si_3$: a polarized neutron diffraction study," J. Phys.: Condens. Matter **14**, 8841–8851 (2002).

[85] L. M. Corliss, N. Elliott, J. M. Hastings, and R. L. Sass, "Magnetic structure of chromium selenide," Phys. Rev. **122**, 1402–1406 (1961).

[86] J R Stewart, G Ehlers, A S Wills, S T Bramwell, and J S Gardner, "Phase transitions, partial disorder and multi-k structures in $Gd_2Ti_2O_6$," J. Phys.: Condens. Matter **16**, L321–L326 (2004).

[87] Olivier Mentré, Matthieu Kauffmann, Ghislaine Ehora, Sylvie Daviero-Minaud, Francis Abraham, and Pascal Roussel, "Structure, dimensionality and magnetism of new cobalt oxyhalides," Solid State Sci. **10**, 471–475 (2008).

[88] S. Baran, A. Szytuła, D. Kaczorowski, and F. Damay, "Magnetic structures in TmPdIn and TmAgSn," J. Alloys **662**, 11–15 (2016).

[89] R. E. Newnham, *Properties of materials: anisotropy, symmetry, structure* (Oxford University Press, 2005).
